# Postoperative lactate trajectories predict in-hospital adverse outcomes and differ by surgical approach in cardiac surgery: a propensity score-matched study

**DOI:** 10.3389/fcvm.2026.1921332

**Published:** 2026-09-17

**Authors:** Duolikun Mutailifu, Abudousaimi Aini, Malike Mutailipu, Zheng Liu, Abudunaibi Maimaitiaili

**Affiliations:** Department of Cardiac Surgery, The First Affiliated Hospital of Xinjiang Medical University, Urumqi, China

**Keywords:** cardiopulmonary bypass, group-based trajectory modelling, lactate trajectory phenotype, postoperative risk stratification, totally thoracoscopic cardiac surgery

## Abstract

**Background:**

Totally thoracoscopic cardiac surgery (TTCS) employs peripheral cardiopulmonary bypass (CPB) with retrograde aortic perfusion, yet whether its net effect on early tissue perfusion is superior to open sternotomy remains uncertain. This study identified postoperative arterial lactate trajectory phenotypes, examined their association with surgical approach, and evaluated their prognostic value for in-hospital adverse outcomes.

**Methods:**

A single-centre, retrospective, propensity score-matched cohort study enrolled 1,264 patients (632 per group). Group-based trajectory modelling (GBTM) was applied to serial arterial lactate concentrations at nine postoperative time points (T0–T120h). Multinomial logistic regression examined surgical approach–trajectory associations. Exploratory path decomposition estimated the indirect contribution of lactate trajectory to the surgical approach–outcome association; a predictive model for major adverse cardiovascular events (MACE) was constructed from early postoperative lactate measurements.

**Results:**

GBTM identified three trajectory phenotypes: Low-Stable (65.8%), Moderate-Resolving (28.6%), and Persistently Elevated (PE; 5.6%). TTCS was independently associated with reduced odds of Moderate-Resolving (adjusted odds ratio [aOR] 0.70, 95% CI 0.54–0.90) and PE membership (aOR 0.27, 95% CI 0.15–0.49), with the protective association more pronounced for PE. PE trajectory independently predicted MACE (aOR 14.73, 95% CI 7.91–27.45) and mechanical ventilation exceeding 48 h (aOR 20.79). Exploratory path decomposition attributed 24.0%–42.3% of the total association to indirect pathways, though estimates were sensitive to unmeasured confounding. T2h lactate independently predicted MACE (aOR 2.18 per mmol/L; AUC 0.697; optimal cut-off 2.45 mmol/L). All five inflammatory and organ-function biomarkers showed trajectory-dependent responses, with inflammatory peaks preceding organ-injury markers.

**Conclusions:**

TTCS significantly reduced membership in high-risk lactate trajectory phenotypes. PE trajectory independently predicted in-hospital adverse outcomes and mirrored the postoperative inflammation–organ injury cascade. A T2h lactate ≥2.45 mmol/L provides a practical early bedside risk-stratification tool.

## Introduction

Conventional median sternotomy with central aortic cannulation for cardiopulmonary bypass (CPB) carries recognised morbidities, including haemorrhage, respiratory complications, and mediastinal infection, that have driven the evolution towards minimally invasive alternatives (1, 2). Totally thoracoscopic cardiac surgery (TTCS) avoids sternotomy by using femoral vessels for peripheral CPB and the right thoracic wall for intracardiac access (3, 4), and is associated with reduced perioperative transfusion requirements, shorter mechanical ventilation, decreased ICU length of stay, and lower rates of wound and mediastinal infection (2, 5). Femoral arterial retrograde perfusion, however, differs fundamentally from central antegrade perfusion and confers a higher stroke risk (1). Both CPB duration and aortic cross-clamp time are substantially prolonged with TTCS (5, 6). Whether the technical advantages of TTCS sufficiently offset the physiological burden of retrograde perfusion and prolonged CPB exposure, and whether this trade-off ultimately confers superior early organ protection over open surgery, remains unresolved (5, 7, 8).

Postoperative lactate elevation after CPB is a sensitive marker of tissue hypoperfusion and oxygen debt that independently predicts in-hospital mortality, ICU readmission, 30-day mortality (9–11), prolonged mechanical ventilation (12), and acute kidney injury (AKI) (13, 14). Conventional assessment relies on single time-point values, peak concentrations, or fixed thresholds, and cannot distinguish rapid clearance from persistent elevation, two patterns with opposing prognostic implications (9, 10). The magnitude of lactate change relative to baseline is a critical predictor of adverse outcomes (11).

Group-based trajectory modelling (GBTM) addresses these limitations by identifying latent subgroups with distinct longitudinal patterns from serial measurements, transforming lactate from a static scalar value into a temporally resolved trajectory phenotype (15). Subgroups defined by this approach demonstrate gradient associations with mortality and organ injury that outperform traditional indices, including initial value, peak concentration, and clearance rate (16–19).

Whether the distinct perfusion strategies of TTCS and open sternotomy differentially shape post-CPB lactate trajectories, and whether trajectory membership mediates their divergent clinical outcomes, has not been systematically examined (5, 7, 8, 16, 17, 19). In a large propensity score-matched cohort, we applied GBTM to identify early post-CPB lactate trajectory phenotypes, quantified the independent association between surgical approach and trajectory membership, and performed exploratory mediation analysis to estimate the contribution of lactate trajectory to the protective effect of TTCS on in-hospital adverse outcomes. The longitudinal profiles of inflammatory and organ function markers, and the predictive performance of early lactate measurements, were further characterised across trajectory groups. We hypothesise that TTCS reduces the probability of high-risk trajectory membership and that such membership independently accounts for a meaningful proportion of its protective effect on in-hospital outcomes.

## Methods

### Study design and study population

This was a single-centre, retrospective, propensity score-matched (PSM) cohort study conducted using the cardiac surgery database of the First Affiliated Hospital of Xinjiang Medical University (2017–2025), approved by the Institutional Review Board (IRB No. K202606-21). Inclusion criteria were: (i) age ≥ 18 years; (ii) elective cardiac surgery at this centre; (iii) postoperative admission to an ICU or coronary care unit (CCU); and (iv) valid arterial blood lactate values at five or more of the nine predefined postoperative time points (T0–T8). Exclusion criteria were: (i) emergency surgery (including aortic dissection); (ii) intraoperative death; and (iii) repeat admission, with the index hospitalisation retained for analysis. Of 6,531 screened patients, 4,405 met eligibility criteria (720 TTCS; 3,685 open surgery), yielding a matched cohort of 1,264 patients (632 per group; Figure 1).

**Figure 1:**
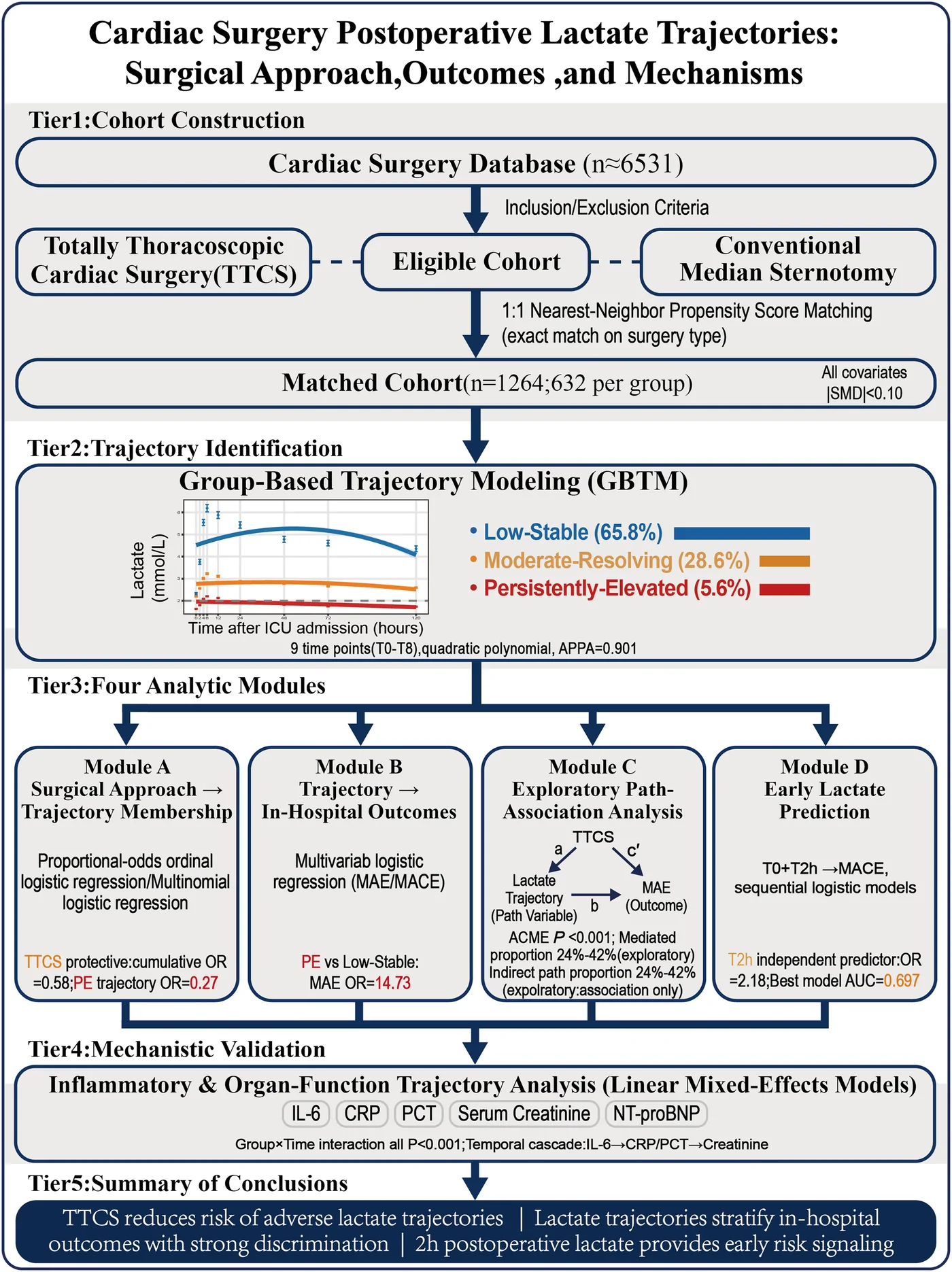
Study design overview. A propensity score–matched cohort (*n* = 1,264; 632 per group) of patients undergoing TTCS or conventional median sternotomy was analysed. Postoperative lactate trajectories were identified by GBTM and subsequently examined across four pre-specified analytic modules. TTCS, totally thoracoscopic cardiac surgery; GBTM, group-based trajectory modeling; MAE, major adverse events; MACE, major adverse cardiovascular events.

### Variable definitions

Surgical approach served as the binary exposure variable. TTCS was defined as a complete intracardiac operation performed under thoracoscopic visualisation through minimally invasive right-thoracic-wall ports, with CPB established via femoral arterial and venous cannulation. The comparator was conventional median sternotomy.

The trajectory modelling inputs comprised arterial blood lactate concentrations at nine predefined postoperative time points (T0–T8, 0–120h), with time expressed as actual elapsed hours throughout. Sampling windows and quality-control criteria are detailed in Supplementary Methods S1.

All PSM covariates were measured preoperatively across four domains: demographics, comorbidities, cardiac function, and laboratory parameters (Supplementary Methods S2). Surgical type (valvular, congenital heart disease, or other) was designated as a mandatory exact-matching variable.

Three composite outcome sets were pre-specified. Major adverse events (MAE) were defined as a composite of in-hospital death, extracorporeal membrane oxygenation (ECMO), renal replacement therapy (RRT) or continuous renal replacement therapy (CRRT), stroke, and intra-aortic balloon pump (IABP) implantation. Encompassing major circulatory, renal, and neurological decompensation events, MAE served as the primary endpoint for trajectory–outcome and pathway association analyses. Major adverse cardiovascular events (MACE) incorporated all MAE components plus low cardiac output syndrome (LCOS), re-intubation, mechanical ventilation exceeding 48 h, and re-exploration for haemostasis; this broader event spectrum enhanced model stability. Defining MACE independently of trajectory modelling precluded circular reasoning (Supplementary Methods S9). MACE-Core retained only severe cardiovascular and cerebrovascular events and served to assess the robustness of predictive conclusions to endpoint specification.

Secondary and continuous outcome definitions are provided in Supplementary Methods S3.

### Propensity score matching

Propensity scores were derived from logistic regression incorporating preoperative covariates, with surgical approach as the outcome. One-to-one nearest-neighbour matching without replacement was then performed under an exact-matching constraint on surgical type. Matching quality was assessed by standardised mean differences (SMDs). Full details of the matching algorithm, caliper specification, and pre- and post-matching statistical comparisons are provided in Supplementary Methods S4.

CPB duration, aortic cross-clamp time, total operative duration, and intraoperative blood loss and transfusion were classified as post-exposure variables and excluded from PS matching to prevent post-treatment bias. Subsequent multivariable analyses comprised an unadjusted model (T-Model 1), estimating the total association, and two stepwise-adjusted models (T-Model 2–3) conditioning on potential mediators to estimate the residual effect. Differences in odds ratios (ORs) across model levels quantified the magnitude of overadjustment.

### Group-based trajectory modelling

Within the matched cohort, GBTM was applied to identify lactate trajectory phenotypes from nine serial postoperative measurements. A three-group quadratic polynomial model was selected on the basis of information criteria, classification quality, and clinical interpretability; details of modelling software, missing-data handling, and group-number selection are provided in Supplementary Methods S5. The three trajectories were named according to their baseline, peak, and terminal lactate levels, and each patient was assigned to a single group by maximum posterior probability. The log-transformed posterior probability of membership in the Persistently Elevated trajectory served as a continuous pathway variable in subsequent exploratory path-association analyses.

### Statistical analysis

Full model specifications, covariate schemes, and sensitivity and subgroup analyses for each analytical step are detailed in Supplementary Methods S6–S10. The association between surgical approach and trajectory membership was pre-specified as the primary estimand and estimated by proportional-odds ordinal logistic regression, treating membership as ordered (Low-Stable < Moderate-Resolving < Persistently Elevated). If a likelihood-ratio test indicated violation of the proportional-odds assumption (*P* < 0.05), multinomial logistic regression, with Low-Stable as the reference category, was pre-specified as the primary analysis; ordinal regression results were retained as a Supplementary reference (S6).

Trajectory–outcome associations used Low-Stable as the reference throughout. Binary outcomes were analysed by logistic regression (reporting adjusted ORs); continuous outcomes were log-transformed and analysed by log-linear regression (reporting geometric mean ratios [GMRs]), with a test for ordinal trend across trajectory groups. Covariates were adjusted in tiers according to the events-per-variable principle. Subgroup analyses and assessments of discrimination and calibration were additionally performed for the primary MAE outcome (S7).

Because the lactate trajectory sampling window (T0–T120h) overlapped temporally with the constituent events of MAE, temporal precedence could not be established. The pathway analysis was therefore restricted to an exploratory path-association analysis; causal mediation inference was not performed. A path decomposition framework was applied to quantify the relative contribution of the indirect pathway operating through lactate trajectory membership. Sensitivity analyses incorporating alternative variable-coding schemes and unmeasured-confounding robustness assessments were conducted (S8).

For early lactate predictive modelling, MACE, defined independently of trajectory modelling, served as the primary outcome to preclude circular reasoning. Five sequential binary logistic models were constructed and evaluated for discrimination (area under the receiver operating characteristic curve [AUC]), calibration, and clinical net benefit by decision curve analysis (DCA). A sensitivity analysis using MACE-Core was performed (S9).

For mechanistic biomarker analyses, linear mixed-effects models, each incorporating a trajectory group × time interaction term, were fitted separately for each of the five inflammatory and organ-function biomarkers. *Post hoc* pairwise comparisons between trajectory groups at each time point were conducted using the emmeans package (S10). All analyses were performed in R version 4.5.2. All tests were two-sided; the significance threshold was *α* = 0.05.

## Results

### Study population and baseline characteristics

The matched cohort comprised 1,264 patients (632 per group). Before matching, key covariates, including age, left ventricular ejection fraction (LVEF), and pulmonary arterial hypertension, were substantially imbalanced (maximum |SMD| = 0.865). After matching, all covariate |SMD| values fell below 0.10 (maximum 0.061), and surgical-type composition was identical between groups (Table 1; Supplementary Figure S1). In the TTCS group, CPB duration (95 vs. 84 min) and aortic cross-clamp time (60 vs. 54 min) were each longer, whereas total operative duration (235 vs. 270 min) and intraoperative transfusion rate (34.8% vs. 48.6%) were lower (all *P* < 0.001).

**Table 1:** Baseline characteristics before and after propensity score matching.

Variable	Before PSM				After PSM			
	TTCS (*n* = 720)	Sternotomy (*n* = 3685)	*P*	SMD	TTCS (*n* = 632)	Sternotomy (*n* = 632)	*P*	SMD
Age (years)	37.77 ± 11.36	48.00 ± 12.26	<0.001	−0.865	38.70 ± 11.12	38.24 ± 11.95	0.375	0.040
Sex [male, *n* (%)]	245 (34.0%)	1355 (50.5%)	<0.001	−0.333	215 (34.0%)	217 (34.3%)	0.952	−0.007
BMI (kg/m^2^)	23.74 ± 4.11	24.91 ± 5.80	<0.001	−0.233	24.06 ± 3.50	24.10 ± 3.98	0.842	−0.011
Hypertension, *n* (%)	32 (4.4%)	484 (18.0%)	<0.001	−0.430	31 (4.9%)	25 (4.0%)	0.451	0.046
Diabetes, *n* (%)	9 (1.2%)	111 (4.1%)	<0.001	−0.178	9 (1.4%)	7 (1.1%)	0.773	0.028
Smoking, *n* (%)	112 (15.6%)	597 (22.2%)	<0.001	−0.171	103 (16.3%)	100 (15.8%)	0.880	0.013
af, *n* (%)	24 (3.3%)	265 (9.9%)	<0.001	−0.263	24 (3.8%)	26 (4.1%)	0.880	−0.016
CAD, *n* (%)	25 (3.5%)	271 (10.1%)	<0.001	−0.263	25 (4.0%)	23 (3.6%)	0.880	0.017
PAH, *n* (%)	299 (41.5%)	513 (19.1%)	<0.001	0.488	238 (37.7%)	233 (36.9%)	0.776	0.016
Renal insufficiency, *n* (%)	0 (0.0%)	20 (0.7%)	0.012	−0.122	0 (0.0%)	0 (0.0%)	–	
LVEF (%)	63.82 ± 3.97	60.87 ± 5.21	<0.001	0.637	63.53 ± 3.72	63.30 ± 4.00	0.211	0.061
Left atrial diameter (mm)	36.00 (32.00–41.00)	41.00 (36.00–46.00)	<0.001	−0.638	36.00 (32.00–41.00)	35.00 (31.00–41.00)	0.207	0.048
NYHA	2.39 ± 0.98	2.29 ± 1.19	0.016	0.095	2.38 ± 1.01	2.39 ± 0.99	0.825	−0.013
NT-proBNP (pg/mL)	197.22 (80.07–442.25)	376.98 (148.00–734.00)	<0.001	−0.292	193.81 (80.18–443.50)	177.00 (74.50–376.98)	0.418	0.036
Serum creatinine (*μ*mol/L)	61.41 (53.23–69.81)	66.98 (56.40–74.28)	<0.001	−0.274	60.95 (53.00–70.00)	59.25 (51.81–68.74)	0.200	0.038
Haemoglobin (g/L)	134.95 ± 17.73	133.49 ± 18.14	0.052	0.081	134.36 ± 17.96	133.84 ± 19.86	0.631	0.027
Albumin (g/L)	40.03 ± 3.16	38.82 ± 3.79	<0.001	0.348	39.97 ± 3.19	39.81 ± 3.66	0.401	0.046
PT-INR	1.05 (1.00–1.11)	1.07 (1.01–1.11)	0.016	−0.117	1.05 (1.00–1.11)	1.05 (1.00–1.11)	0.911	0.003
Operation type, *n* (%)			<0.001				1.000	
Valve	180 (25.0%)	1901 (70.8%)			175 (27.7%)	175 (27.7%)		
CHD	499 (69.3%)	633 (23.6%)			417 (66.0%)	417 (66.0%)		
Other	41 (5.7%)	151 (5.6%)			40 (6.3%)	40 (6.3%)		

Data are mean ± SD, median (IQR), or *n* (%). Pre-PSM: independent t-test, Mann–Whitney U test, chi-squared test, or Fisher's exact test. Post-PSM: paired t-test, Wilcoxon signed-rank test, or McNemar's test. Matching: 1:1 nearest-neighbor with exact constraint on operation type (caliper = 0.2).

AF, atrial fibrillation; BMI, body mass index; CAD, coronary artery disease; CHD, congenital heart disease; LVEF, left ventricular ejection fraction; NT-proBNP, N-terminal pro-B-type natriuretic peptide; NYHA, New York Heart Association; PAH, pulmonary arterial hypertension; PSM, propensity score matching; SMD, standardized mean difference.

### Identification of three postoperative arterial lactate trajectory phenotypes

In the overall matched cohort, arterial lactate followed a rise-and-fall pattern, with a mean of 1.83 mmol/L at ICU admission, a peak of 2.72 mmol/L at 6 h, and a return to 2.12 mmol/L by 120 h (Supplementary Table S2). GBTM identified a three-group quadratic polynomial model as optimal; classification quality was satisfactory (average posterior probability of assignment [APPA] = 0.901, odds of correct classification [OCC] = 12.66, entropy = 0.883; Supplementary Tables S1, S2; Supplementary Figure S2). Three trajectory phenotypes were characterised by their baseline, peak, and terminal lactate profiles (Figure 2). The Low-Stable trajectory (*n* = 832, 65.8%) remained at approximately 2.0 mmol/L throughout. Comprising 28.6% of patients (*n* = 361), the Moderate-Resolving trajectory peaked at 2.84 mmol/L at approximately 40 h before declining. The Persistently Elevated (PE) trajectory (*n* = 71, 5.6%) entered the ICU at 4.52 mmol/L, peaked at 5.27 mmol/L at approximately 53 h, and remained elevated at 4.08 mmol/L at 120 h.

**Figure 2:**
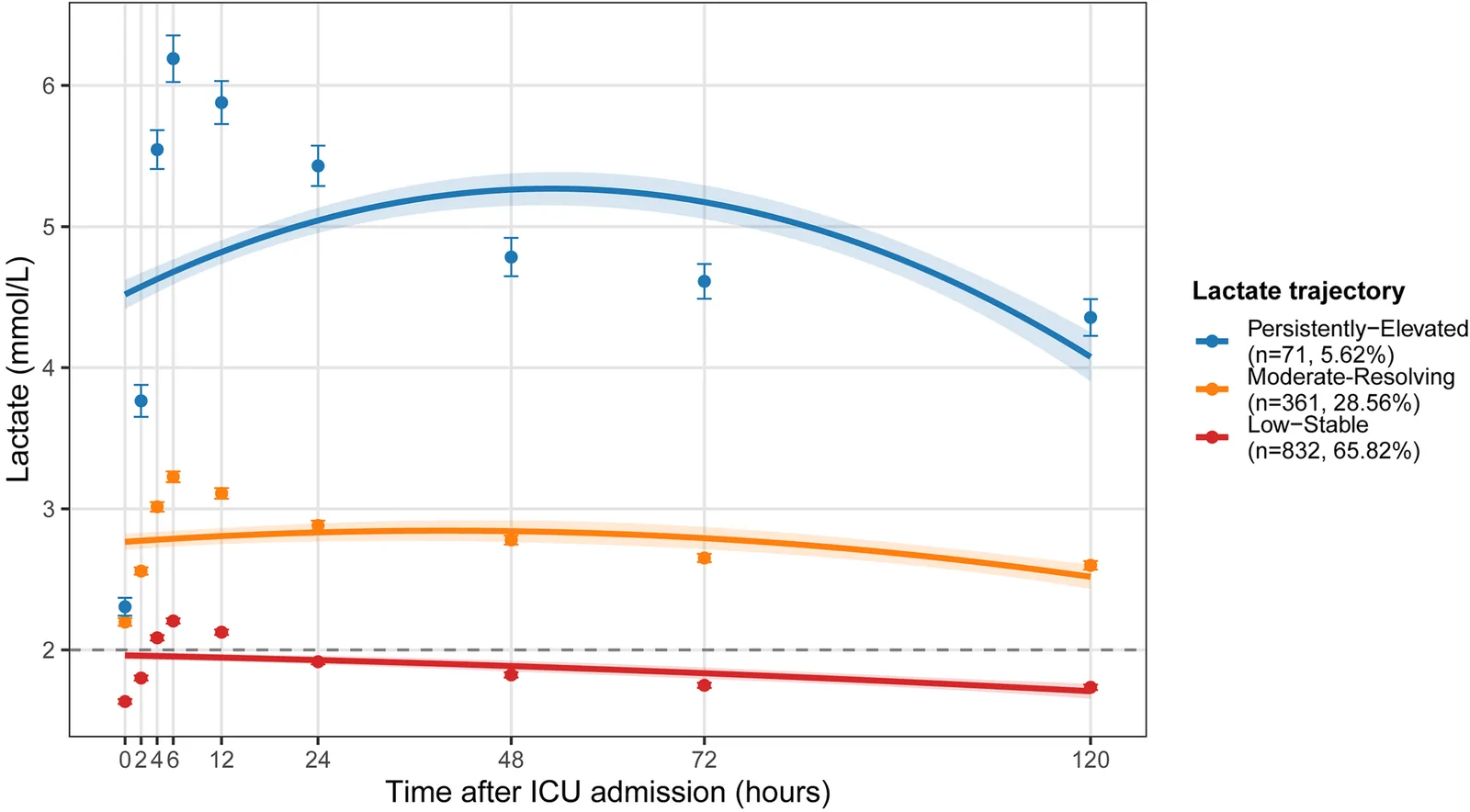
Three postoperative arterial lactate trajectory phenotypes identified by group-based trajectory modeling (k = 3). Propensity-matched cohort (*n* = 1,264): Low-Stable (65.8%), Moderate-Resolving (28.6%), and Persistently-Elevated (5.6%). Solid lines, fitted quadratic trajectories (95% CI shading); points, observed group means ± SE. Dashed line, 2.0 mmol/L. APPA = 0.901; entropy = 0.883.

### Association between surgical approach and lactate trajectory membership

Trajectory membership distributions differed significantly between surgical groups (Figure 3). In the TTCS group, 72.2%, 25.3%, and 2.5% of patients belonged to the Low-Stable, Moderate-Resolving, and PE trajectories, respectively, compared with 59.5%, 31.8%, and 8.7% in the open sternotomy group (*χ*^2^ = 33.77, *P* < 0.001). Because the likelihood-ratio test indicated violation of the proportional-odds assumption (*P* = 0.038), multinomial logistic regression with Low-Stable as the reference category was adopted as the primary analysis per the pre-specified protocol. TTCS was independently associated with reduced odds of Moderate-Resolving membership (aOR 0.70, 95% CI: 0.54–0.90, *P* = 0.006) and, more substantially, with PE membership (aOR 0.27, 95% CI: 0.15–0.49, *P* < 0.001), reflecting a graded protective association across increasing trajectory severity (Table 2). Supplementary ordinal logistic regression yielded cumulative ORs of 0.54–0.58 across T-Model tiers (all *P* < 0.001; Supplementary Table S3); binary PE-specific analysis confirmed stable ORs of 0.27–0.28 (all *P* < 0.001; Supplementary Table S4), supporting estimate stability across modelling approaches. Subgroup analyses stratified by surgical type showed consistent protective directionality, with no significant treatment–subgroup interaction (*P* for interaction = 0.163; Supplementary Table S5). The protective effect was most pronounced in the CHD subgroup (OR 0.21, 95% CI: 0.09–0.44, *P* < 0.001; event rates: 2.4% [10/417] vs. 10.3% [43/417]) and directionally consistent, though not statistically significant, in the valvular subgroup (OR 0.35, 95% CI: 0.08–1.21, *P* = 0.111; event rates: 2.3% [4/175] vs. 6.3% [11/175]).

**Figure 3:**
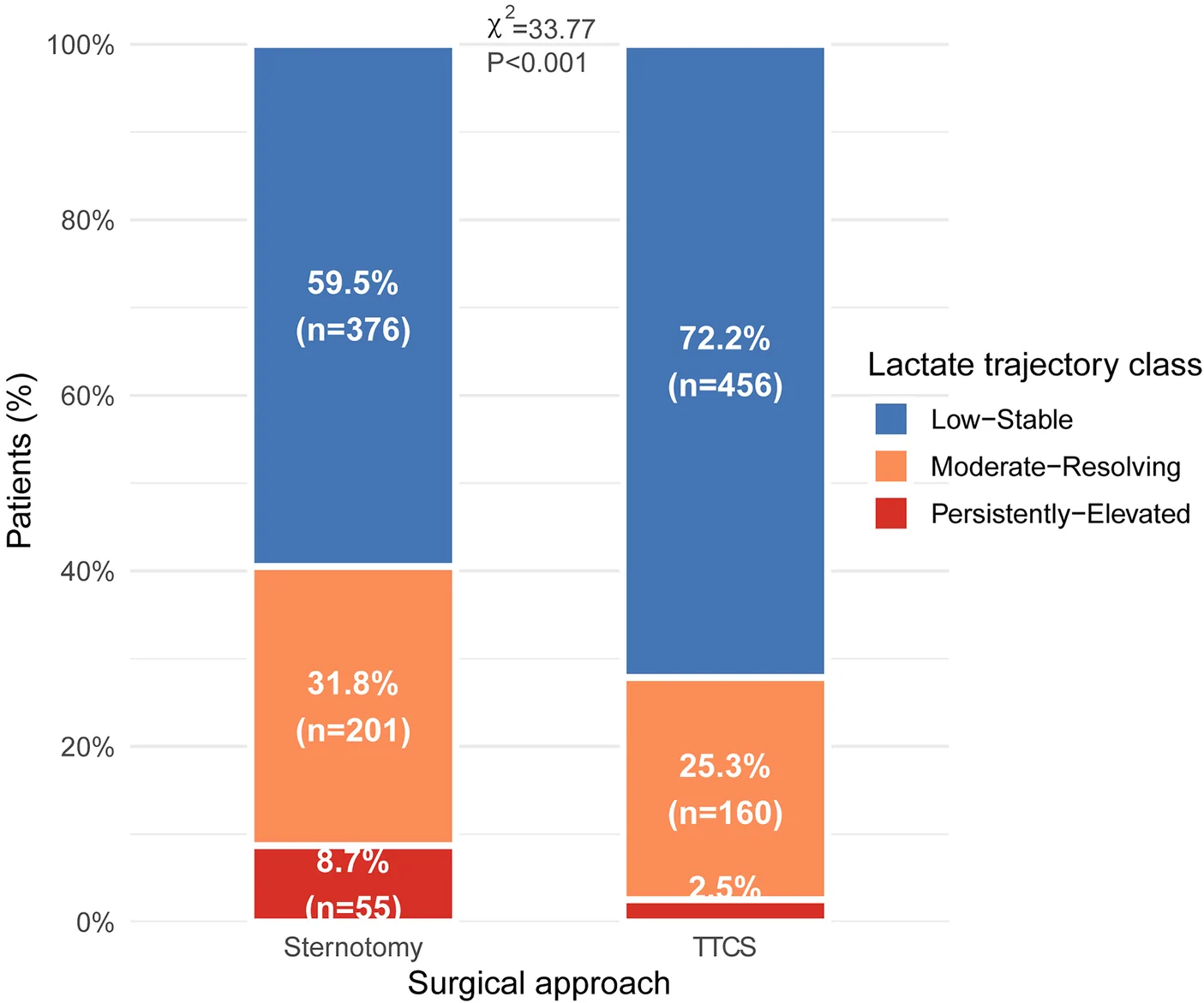
Lactate trajectory distribution by surgical approach. Proportions of three trajectory phenotypes in the sternotomy and TTCS groups (*n* = 632 per group); numbers within bars indicate patient counts. *χ*^2^ = 33.77, *P* < 0.001. TTCS, total thoracoscopic cardiac surgery.

**Table 2:** Association between surgical approach and postoperative lactate trajectory membership.

T-Model	Contrast	OR	LCI	UCI	OR	*P*
T-Model 1 (Unadjusted)	Moderate-Resolving vs. Low-Stable	0.656373	0.511938	0.841557	0.66 (0.51−0.84)	0.000899
T-Model 1 (Unadjusted)	Persistently-Elevated vs. Low-Stable	0.239766	0.135145	0.425378	0.24 (0.14–0.43)	1.05E-06
T-Model 2 (Primary adjusted)	Moderate-Resolving vs. Low-Stable	0.695455	0.536433	0.901619	0.70 (0.54–0.90)	0.00611
T-Model 2 (Primary adjusted)	Persistently-Elevated vs. Low-Stable	0.272259	0.150454	0.492675	0.27 (0.15–0.49)	1.71E-05
T-Model 3 (Fully adjusted)	Moderate-Resolving vs. Low-Stable	0.687433	0.529175	0.893019	0.69 (0.53–0.89)	0.00499
T-Model 3 (Fully adjusted)	Persistently-Elevated vs. Low-Stable	0.267235	0.147907	0.482834	0.27 (0.15–0.48)	1.23E-05

Multinomial logistic regression; reference category: Low-Stable. ORs represent the odds of TTCS versus open sternotomy. Proportional-odds assumption violated (LRT *P* = 0.038); multinomial regression adopted as primary analysis. T-Model 1: unadjusted; T-Model 2: adjusted for key preoperative covariates; T-Model 3: fully adjusted for all preoperative covariates.

CI, confidence interval; LRT, likelihood-ratio test; OR, odds ratio.

### Association between lactate trajectory and in-hospital adverse outcomes

In the matched cohort, 102 MAE occurred (8.1%). All nine in-hospital adverse outcome rates increased in a stepwise fashion across trajectory groups (all *P* < 0.001; Figure 4A; Supplementary Table S6), with the steepest gradients observed for MAE (3.5% → 12.7% → 38.0%) and mechanical ventilation exceeding 48 h (4.7% → 17.7% → 50.7%). ICU and total hospital lengths of stay also increased progressively with trajectory severity (Supplementary Figure S3).

**Figure 4:**
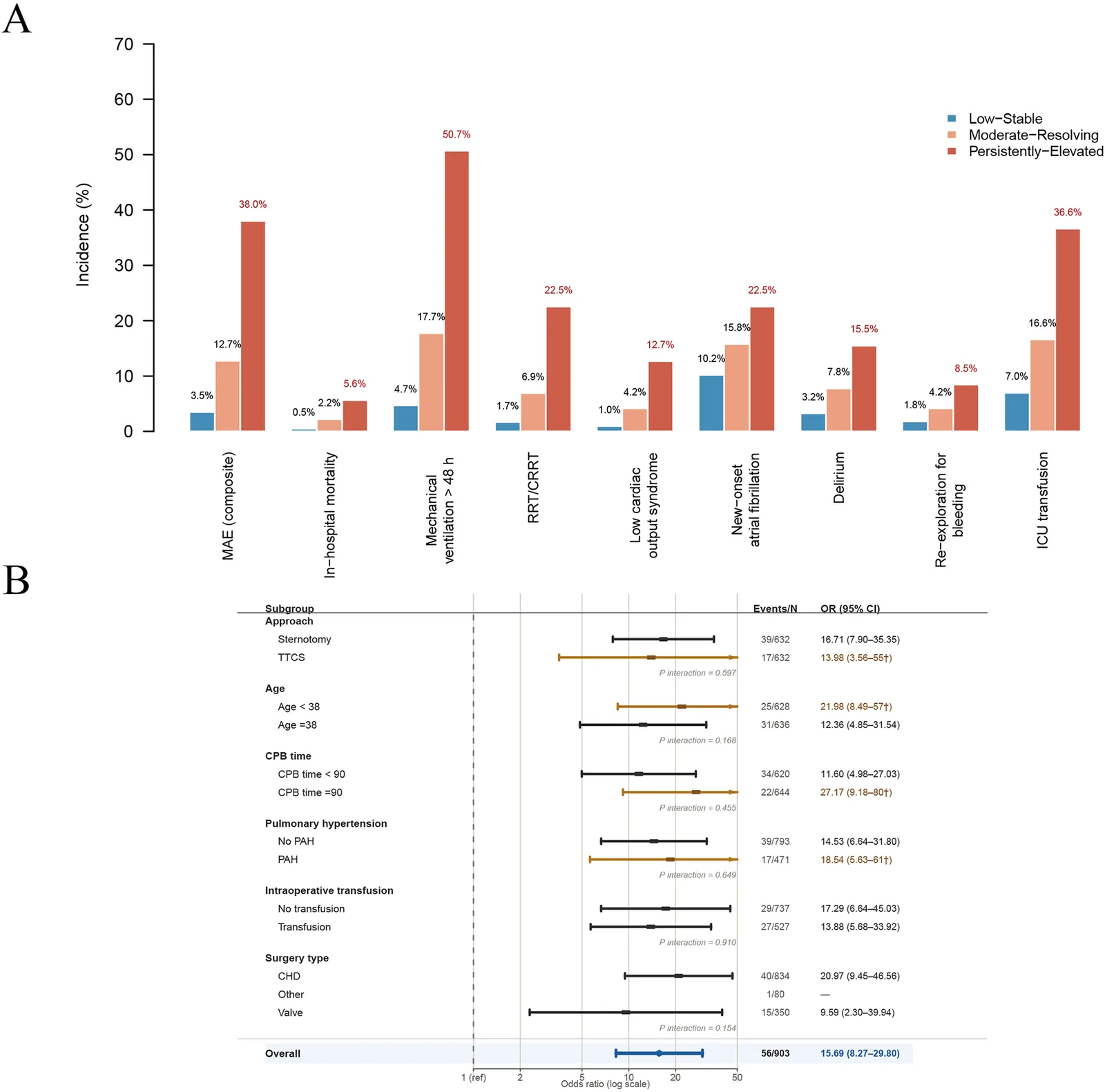
In-hospital outcomes and subgroup consistency. **(A)** Incidence of nine adverse outcomes by trajectory class; all increased stepwise from Low-Stable to Persistently-Elevated (all *P* < 0.001). **(B)** Forest plot of adjusted ORs (95% CI, log scale) for Persistently-Elevated trajectory membership and MAE across prespecified subgroups (Low-Stable as reference); no significant treatment–subgroup interaction was detected (all *P* for interaction > 0.05). †Upper CI limit truncated (>50). MAE, major adverse events; RRT/CRRT, renal replacement therapy/continuous renal replacement therapy; CPB, cardiopulmonary bypass; PAH, pulmonary arterial hypertension; CHD, congenital heart disease; TTCS, total thoracoscopic cardiac surgery.

After multivariable adjustment, PE trajectory membership retained strong independent associations with all primary outcomes relative to Low-Stable membership, with the largest effect sizes observed for MAE (aOR 14.73, 95% CI 7.91–27.45), mechanical ventilation exceeding 48 h (aOR 20.79), RRT/CRRT (aOR 13.96), and in-hospital mortality (aOR 9.11, *P* = 0.002); Moderate-Resolving membership showed associations of intermediate magnitude (MAE: aOR 3.79). Adjusted geometric mean ratios for the three continuous time-based outcomes in the PE vs. Low-Stable group were 3.47, 1.34, and 1.07, respectively (all *P* < 0.05; Supplementary Table S8). Subgroup analyses revealed consistent directionality of the PE trajectory effect on MAE across all strata (aORs 9.59–27.17; *P* for interaction 0.154–0.910; Figure 4B). The primary model showed satisfactory discrimination and calibration (C-statistic 0.770, Nagelkerke *R*^2^ = 0.225, Hosmer–Lemeshow *P* = 0.445).

### Exploratory path association analysis: decomposition of the lactate trajectory contribution to the protective effect of totally thoracoscopic cardiac surgery

The total association of TTCS with MAE was estimated at aOR 0.47 (95% CI 0.30–0.75, *P* = 0.001; Supplementary Table S9). As temporal precedence between lactate trajectory assignment and MAE component events could not be established, all decomposition results are reported as exploratory association findings; no causal mediation inference is drawn.

On the linear probability scale, bootstrap path decomposition yielded a total effect of −0.042 (*P* = 0.010; Table 3; Figure 5), with an indirect-path estimate of −0.010 (95% CI −0.016 to −0.005, *P* < 0.001) and a direct-path estimate of −0.032 (95% CI −0.061 to −0.001, *P* = 0.042). Sensitivity analyses using ordinal and binary trajectory coding gave directionally consistent indirect paths (indirect-pathway proportions 42.3% and 32.0%; 24%–42% across the three coding schemes). Because the proportion mediated is a ratio of two bootstrapped quantities, its interval is not bounded to [0%, 100%]; under ordinal coding the direct path was indistinguishable from zero (95% CI −0.056–0.004), and resamples with a sign-reversed direct path inflated the upper limit to 122.0% (Table 3 footnote). All proportions are exploratory.

**Table 3:** Exploratory path-analytic decomposition: contribution of lactate trajectory to the protective effect of TTCS on MAE.

Model	Effect	Estimate (95% CI)	*P* value
Main: log(posterior_PE)	Indirect path	−0.010 (−0.016 to −0.005)	<0.001
Main: log(posterior_PE)	Direct path	−0.032 (−0.061 to −0.001)	0.0416
Main: log(posterior_PE)	Total Effect	−0.042 (−0.072 to −0.011)	0.0104
Main: log(posterior_PE)	Indirect/Total	24.0% (10.2% to 81.0%)	0.0104
Sens1: ordinal trajectory	Indirect path	−0.019 (−0.029 to −0.007)	<0.001
Sens1: ordinal trajectory	Direct path	−0.026 (−0.056 to 0.004)	0.0924
Sens1: ordinal trajectory	Total Effect	−0.045 (−0.074 to −0.011)	0.0084
Sens1: ordinal trajectory	Indirect/Total	42.3% (15.2% to 122.0%)	0.0084
Sens2: binary high-risk traj.	Indirect path	−0.016 (−0.028 to −0.006)	0.0012
Sens2: binary high-risk traj.	Direct path	−0.034 (−0.062 to −0.003)	0.0316
Sens2: binary high-risk traj.	Total Effect	−0.050 (−0.079 to −0.017)	0.0016
Sens2: binary high-risk traj.	Indirect/Total	32.0% (12.3% to 84.5%)	0.0028

Estimates on the linear probability scale; CIs derived by bootstrap resampling. Temporal overlap between lactate trajectory assessment (T0–T120h) and MAE precluded causal inference; all findings are exploratory. Indirect/Total: proportion of total effect attributed to the indirect pathway. As this proportion is a ratio of two bootstrapped estimates, its interval is not constrained to [0%, 100%]; the Sens1 upper limit of 122.0% arises from resamples in which the near-null direct path (95% CI −0.056–0.004) reversed sign, and does not denote a genuine proportion exceeding unity. The absolute indirect- and direct-path estimates are the primary metric. Pathway variable coding — Main: log-transformed posterior probability of PE trajectory; Sens1: ordinal trajectory score (0–2); Sens2: binary high-risk indicator (PE vs. non-PE).

CI, confidence interval; MAE, major adverse events; PE, persistently elevated trajectory.

**Figure 5:**
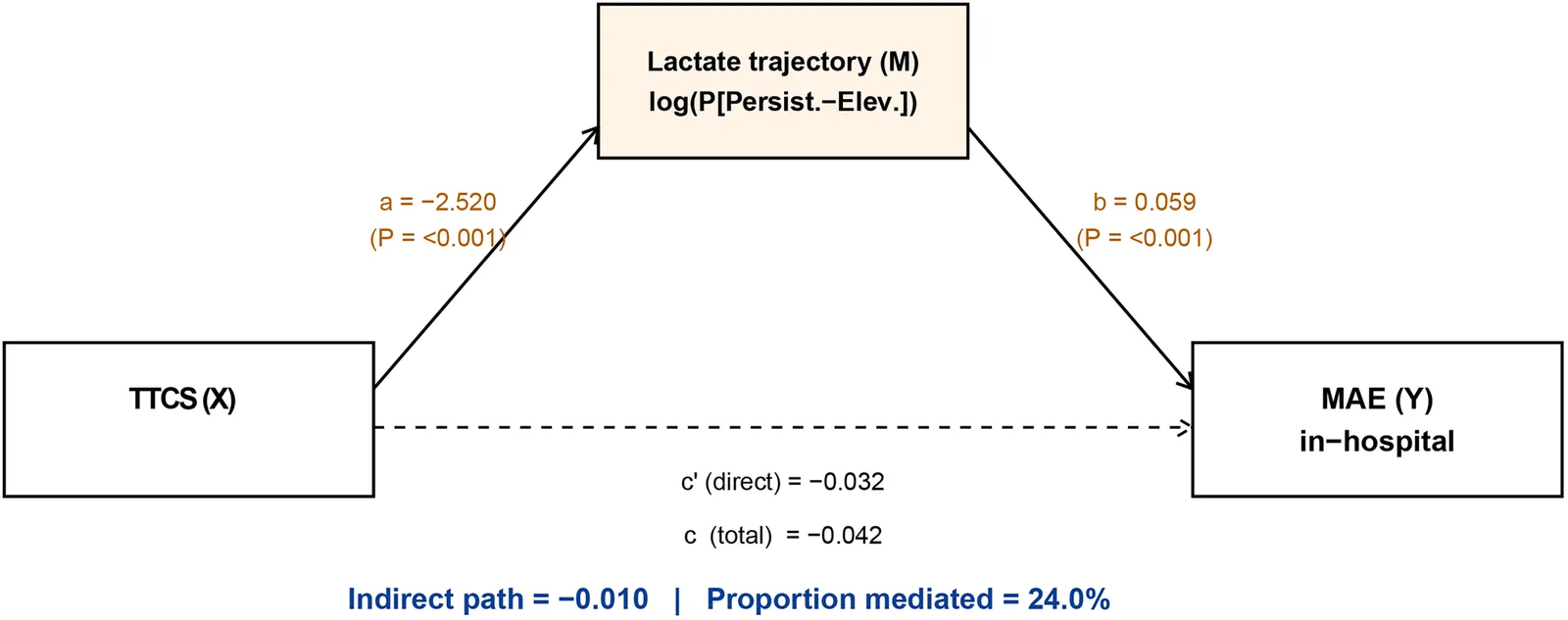
Exploratory path decomposition of the association between TTCS and MAE via the persistently-elevated lactate trajectory. Path coefficients estimated on the linear probability scale (bootstrap, *n* = 1,000 iterations). Indirect-path estimate (a × b): −0.010 (95% CI: −0.016 to −0.005), representing 24.0% of the total effect (c = −0.042); direct-path estimate (c′): −0.032 (95% CI: −0.061 to −0.001). Temporal precedence between trajectory assignment and MAE could not be established; all estimates reflect statistical association decomposition only. MAE, major adverse events; PE, persistently elevated; TTCS, total thoracoscopic cardiac surgery.

Robustness analyses indicated that the critical confounding correlation ρ* required to nullify the ACME was only 0.05–0.10 (Supplementary Figure S4), a threshold readily exceeded by unmeasured confounders such as baseline haemodynamic status in the post-cardiac-surgery ICU setting. All path decomposition estimates should therefore be regarded collectively as exploratory association signals.

### Predictive value of early lactate (T0 and T2h) for major adverse cardiovascular events

Among 1,254 patients with complete early lactate data, MACE occurred in 274 (21.9%) and MACE-Core in 128 (10.2%). T2h lactate was significantly higher in patients who subsequently developed MACE than in those who did not (median 2.40 vs. 2.00 mmol/L, *P* < 0.001). T0 lactate and ΔLactate (= T2h−T0) showed consistent directional differences (Supplementary Figure S5).

Sequential model construction (Table 4; Figure 6A) revealed that T2h lactate alone (P-Model 2, AUC 0.665) outperformed T0 lactate alone (P-Model 1, AUC 0.609; DeLong *P* = 0.007), whilst combining both time points (P-Model 3, AUC 0.671) yielded no significant incremental gain over T2h alone (*P* = 0.179). Discrimination improved with successive inclusion of preoperative covariates (P-Model 4, AUC 0.682) and surgical approach and type (P-Model 5, AUC 0.697, 95% CI 0.661–0.733; fivefold cross-validated AUC 0.680). In the fully adjusted P-Model 5, T2h lactate remained an independent predictor of MACE (aOR 2.18 per mmol/L, 95% CI 1.76–2.71, *P* < 0.001), whilst T0 lactate did not attain independent significance (aOR 1.25, *P* = 0.120); TTCS was independently protective (aOR 0.60, *P* < 0.001). P-Model 5 demonstrated satisfactory calibration (Hosmer–Lemeshow *P* = 0.080) and positive net benefit across threshold probabilities of 5%–40% (Figures 6B, C). The Youden-optimal cutoff for P-Model 2 was a T2h lactate of 2.45 mmol/L. For P-Model 5, an optimal probability threshold of 0.212 yielded sensitivity of 66.4%, specificity of 65.6%, and NPV of 87.5% (Table 4). Sensitivity analyses restricted to MACE-Core confirmed consistent findings (AUC 0.684; Supplementary Tables S13, S14).

**Table 4:** Discrimination and classification performance of sequential early-lactate predictive models for MACE.

P-Model	AUC	AUC_LCI	AUC_UCI	AUC_text	Threshold	Sensitivity	Specificity	PPV	NPV	Accuracy	Youden_J
P-Model 1: T0 alone	0.6095	0.5718	0.6472	0.609 (0.572−.647)	0.2133	0.635	0.5398	0.2784	0.841	0.5606	0.1748
P-Model 2: T2h alone	0.6655	0.6269	0.7041	0.665 (0.627−.704)	0.2505	0.4964	0.7735	0.3799	0.846	0.7129	0.2698
P-Model 3: T0 + T2h	0.671	0.6325	0.7094	0.671 (0.633−.709)	0.2502	0.5219	0.7663	0.3844	0.8515	0.7129	0.2882
P-Model 4: T0 + T2h + Preop	0.682	0.6451	0.7188	0.682 (0.645−.719)	0.2447	0.5219	0.7459	0.3648	0.848	0.697	0.2678
P-Model 5: + VATS + Surgery	0.6967	0.6606	0.7328	0.697 (0.661−.733)	0.2123	0.6642	0.6551	0.35	0.8747	0.6571	0.3193

Probability thresholds selected by maximizing the Youden index (J = sensitivity + specificity−1). The T2h lactate cutoff of 2.45 mmol/L for P-Model 2 was back-calculated from the Youden-optimal probability threshold via the univariable logistic regression equation.

AUC, area under the ROC curve; MACE, major adverse cardiovascular events; NPV, negative predictive value; PPV, positive predictive value.

**Figure 6:**
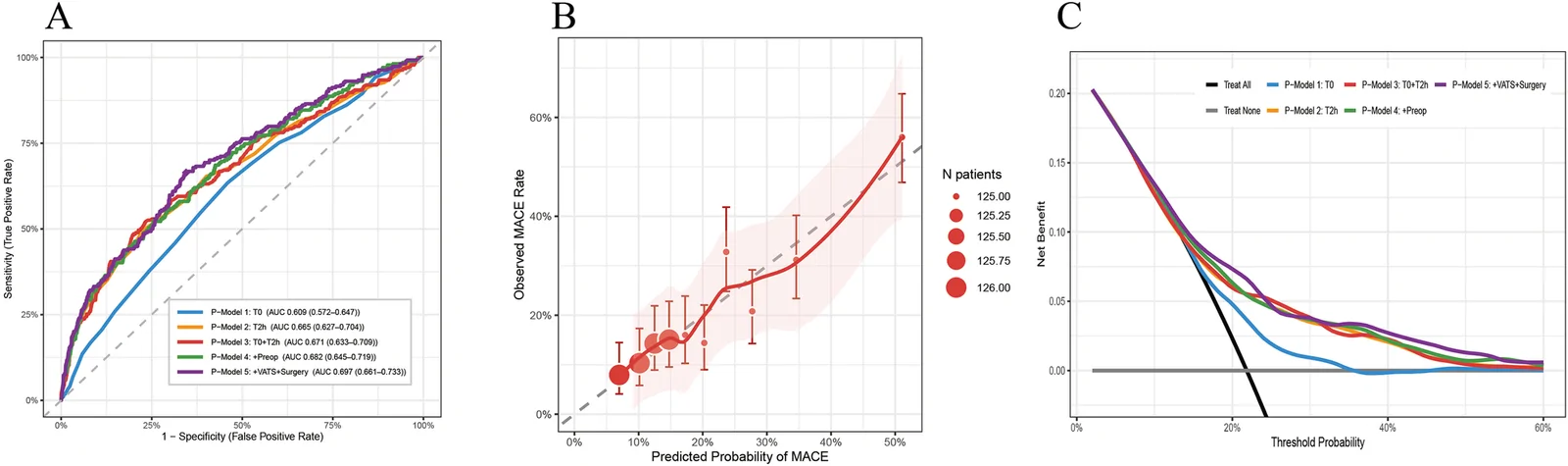
Predictive performance of early postoperative lactate for MACE. **(A)** ROC curves for five sequential models (P-Models 1–5). Optimal threshold for P-Model 5 (0.212, Youden index): sensitivity 66.4%, specificity 65.6%. **(B)** Calibration plot for P-Model 5; bubbles represent predicted-probability deciles (area ∝ patient count); Hosmer–Lemeshow *P* = 0.080. **(C)** Decision curve analysis; all models provided positive net benefit across threshold probabilities of 5%–40%. AUC, area under the curve; MACE, major adverse cardiovascular events; T0, ICU-admission lactate; T2h, 2-hour postoperative lactate; TTCS, total thoracoscopic cardiac surgery.

### Lactate trajectory and postoperative inflammatory and organ function responses

Linear mixed-effects models revealed significant trajectory group × time interaction effects for all five biomarkers (*P* for interaction < 0.001), confirming a trajectory-stratified response that persisted across the postoperative course, with the PE group consistently at the apex. Group main effects were significant for IL-6, CRP, and NT-proBNP (all *P* ≤ 0.002); between-group differences for PCT and creatinine, though not significant as main effects, widened progressively over time (Supplementary Table S15; Figure 7). At peak, IL-6 and PCT concentrations in the PE group were approximately 5.5- and 5.3-fold those of the Low-Stable group, respectively, whilst CRP and NT-proBNP were 2.3- to 3.5-fold higher; the Moderate-Resolving group remained consistently intermediate. Serum creatinine in the PE group rose progressively from POD 1, peaking at approximately 189 μmol/L at POD 3–4 without returning towards baseline by POD 5, in contrast to the transient, mild elevations observed in the other two groups. Peak concentrations across all markers followed an ordered temporal cascade: IL-6 (POD 1) → CRP, PCT, and NT-proBNP (POD 2–3) → creatinine (POD 3–4).

**Figure 7:**
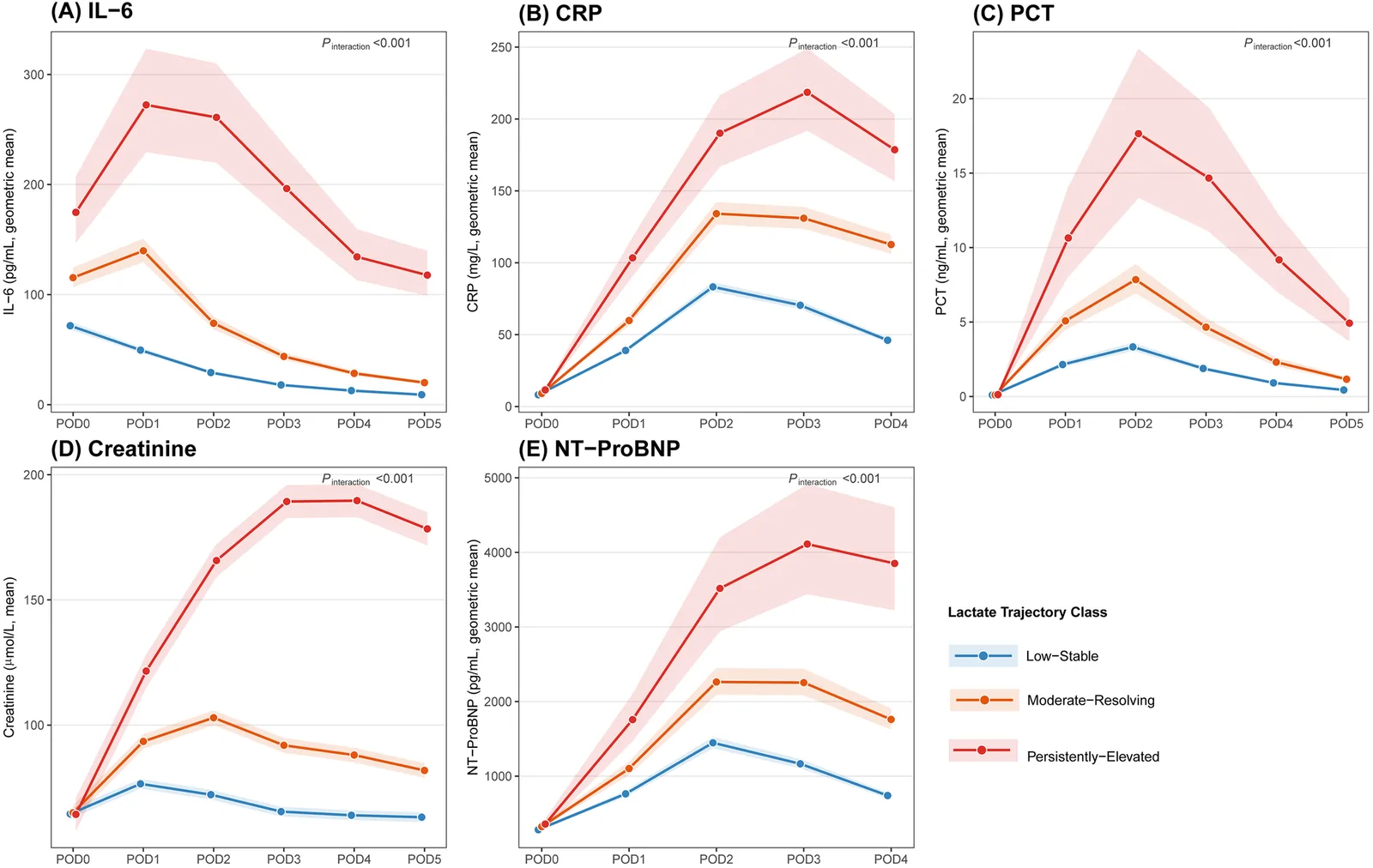
Postoperative biomarker trajectories stratified by lactate class. Estimated marginal means (±95% CI) from linear mixed-effects models adjusted for age and LVEF. Panels A–E display IL-6, CRP, PCT, serum creatinine, and NT-proBNP, respectively; IL-6, CRP, PCT, and NT-proBNP presented as geometric means (log-transformed), and creatinine as arithmetic mean. The Persistently-Elevated group showed the highest peak concentrations and slowest resolution across all biomarkers; peak timing followed the sequence: IL-6 (POD 1) → CRP, PCT, NT-proBNP (POD 2–3) → creatinine (POD 3–4). All trajectory × time interactions: P < 0.001. CRP, C-reactive protein; IL-6, interleukin-6; LVEF, left ventricular ejection fraction; NT-proBNP, N-terminal pro-B-type natriuretic peptide; PCT, procalcitonin; POD, postoperative day.

## Discussion

The present study revealed a physiological paradox. Despite longer CPB duration and aortic cross-clamp time in the TTCS group, the adjusted odds of membership in the high-risk PE trajectory were approximately one-third those of the open sternotomy group (aOR 0.27). This dissociation indicates that net postoperative tissue oxygen-metabolic balance is shaped by multiple interacting factors beyond CPB duration alone. The differential perioperative exposure profile of TTCS, including shorter total operative duration and lower intraoperative transfusion requirements, may partly account for this net protection, consistent with published evidence that TTCS yields the lowest blood loss and transfusion requirements among minimally invasive approaches (2, 5, 6, 8, 20).

Effect estimates remained stable across all adjustment tiers (Supplementary Tables S3, S4), indicating that over-adjustment bias from conditioning on CPB duration and transfusion volume was minimal. The protective effect of TTCS was therefore not principally mediated through these two variables, a conclusion corroborated by the predominance of the direct pathway in the path decomposition analysis.

Concerns have been raised that peripheral femoral cannulation for retrograde perfusion may alter vascular shear stress and promote plaque embolisation (1). Serial lactate data from the present study argue against this concern: TTCS patients were disproportionately assigned to low-risk trajectories, with no evidence of systemic tissue oxygenation impairment. Under standardised patient selection and operative technique, peripheral retrograde perfusion does not appear to confer greater systemic tissue oxygen debt than central aortic cannulation, at least as indexed by postoperative arterial lactate trajectories.

Conventional post-cardiac-surgery lactate assessment relies on static indices, including fixed time-point peak values, absolute thresholds (e.g., >4 mmol/L), and clearance rates (9, 10), yet none of these distinguishes between temporal patterns with markedly different prognostic implications. GBTM identified three metabolically distinct trajectory phenotypes in the present cohort. The PE subgroup, comprising only 5.6% of patients, maintained persistently elevated concentrations from ICU admission onward. A conventional threshold-based approach would have subsumed these patients within the broad hyperlactataemia category, thereby obscuring their distinct clinical identity. Trajectory modelling thus provides an independent dimension of phenotypic recognition for this extreme-risk subgroup, a finding in keeping with reports across comparable critically ill populations. In sepsis-associated acute respiratory distress syndrome, ICU sepsis, and critical hyperlactataemia, persistently elevated lactate subgroups consistently carry the worst prognosis (16–19).

The protective effect of TTCS followed a severity-dependent gradient, with an adjusted OR of 0.27 for PE membership compared with 0.70 for Moderate-Resolving membership. This pattern is consistent with preferential attenuation of the most severe perfusion injury, a directional effect that held across all surgical-type subgroups (*P* for interaction = 0.163).

The pronounced dose–response gradient between lactate trajectory phenotype and in-hospital adverse outcomes (PE trajectory: adjusted OR for MAE = 14.73) provided structural support for the b-path of the TTCS → lactate trajectory → MAE mediation framework. However, temporal precedence could not be established, as the lactate sampling window (T0–T120h) overlapped with MAE component events; all decomposition analyses were therefore designated exploratory. Although the indirect pathway accounted for 24%–42% of the total effect across three coding schemes (Table 3; Figure 5), these estimates were highly sensitive to unmeasured confounding, and even at the upper bound, more than half of the protective association operated through direct pathways bypassing lactate trajectory. One such parallel mechanism involves transfusion: as an independent risk factor for hyperlactatemia (9, 10), the lower transfusion rate with TTCS may directly reduce the probability of high-risk trajectory membership. Mechanistically, lactate is a downstream product of aerobic glycolysis and tissue hypoxia (21, 22), so its association with adverse outcomes may be driven predominantly by shared upstream pathology: myocardial hypoperfusion and microcirculatory dysfunction. Whether lactate trajectory is a passive epiphenomenon or a causal mediator therefore cannot be resolved within an observational framework.

Lactate trajectory differences were not isolated phenomena but were embedded within the CPB-activated SIRS cascade (23). IL-6 serves as the central regulatory mediator of this cascade, driving the inflammation–organ injury continuum, and all five biomarkers exhibited an ordered temporal sequence concordant with trajectory severity, with systemic inflammation preceding organ injury. This sequence corroborates the established independent association between hyperlactatemia and postoperative AKI, which manifests as delayed creatinine elevation (14). Persistent IL-6 and CRP elevation is an independent predictor of postoperative mortality (24), and sustained SIRS substantially increases the risk of new-onset AF, stroke, and death (25). These associations are consistent with the 38.0% MAE rate observed in the PE group. Published mechanistic evidence indicates that lactate may activate endothelial NF-*κ*B via MCTs and upregulate pro-inflammatory genes (21), whilst activated inflammatory cells concurrently shift towards aerobic glycolysis, generating further lactate (22), potentially establishing a self-reinforcing loop. Elevated lactate may additionally impair tubular acid-base regulation, compounding AKI risk (13, 26). Within the present observational framework, these mechanisms provide biological plausibility for the PE trajectory's co-occurrence with the inflammation–organ injury cascade and for the exploratory path-association findings described above; whether lactate trajectory is a causal participant or a parallel indicator cannot be established from the current data.

From a clinical translation standpoint, T2h lactate outperformed T0 lactate in predicting MACE. After multivariable adjustment, T0 lactate was no longer independently significant, whereas T2h lactate remained independently associated with outcome (adjusted OR 2.18 per 1 mmol/L increment).

Compared with the 24-h postoperative or preoperative time points reported in prior literature (26), this finding advances the effective prediction window to 2 h postoperatively, potentially enabling earlier risk-directed clinical assessment. A T2h lactate cut-off of 2.45 mmol/L was identified by applying the Youden index to P-Model 2 (univariable logistic regression; method detailed in Supplementary Methods S9 and the Table 4 footnotes). Risk stratification at this threshold is achievable at the bedside using routine arterial blood gas analysis at ICU admission, rendering the approach operationally feasible. Although P-Model 5 (AUC 0.697) demonstrated lower discrimination than published nomograms incorporating additional preoperative and intraoperative variables (AUC 0.799–0.802) (9, 10, 14), the required assessment time points for those nomograms extend well beyond 2 h postoperatively. T2h lactate should therefore be regarded as an early complement to existing risk-stratification instruments rather than a substitute. Given the gradient-protective association of TTCS with PE trajectory membership observed in this study, surgical approach may represent a modifiable upstream factor in postoperative risk trajectory when anatomical and clinical conditions permit, a hypothesis that warrants prospective validation.

This study has several limitations. First, the single-centre, propensity score-matched observational design cannot exclude residual confounding or indication bias. Second, trajectory membership was assigned deterministically by maximum posterior probability and treated as a categorical variable; this approach may underestimate the variance of effect estimates, although the high classification quality (APPA = 0.901) suggests the resulting bias is small. Third, temporal overlap between the lactate sampling window and MAE component events precluded causal inference; path-association estimates were highly sensitive to unmeasured confounding (critical ρ* 0.05–0.10) and should be regarded as exploratory. Fourth, the cohort was enriched for congenital heart disease and is thus younger and less atherosclerotic than typical adult cardiac surgery populations; as totally thoracoscopic cardiac surgery excludes coronary revascularisation, the Persistently Elevated prevalence (5.6%), the phenotype distribution, and the 2.45 mmol/L T2h cut-off are conditioned on this case-mix and should be regarded as centre-specific, requiring recalibration before application to older, coronary- or valve-predominant cohorts. Finally, the PE subgroup was small (*n* = 71), the predictive model achieved only moderate discrimination (AUC 0.697), follow-up was confined to in-hospital outcomes. The trajectory classification scheme, predictive cut-off, and exploratory path-association findings therefore all require prospective validation in independent external cohorts; extrapolation beyond the present population warrants caution.

## Data Availability

The original contributions presented in the study are included in the article/Supplementary Material, further inquiries can be directed to the corresponding author.

## References

[1] Werner P Winter M Coti I Kahrovic A Andreas M Haberl T. State-of-the-art review: advantages and disadvantages of femoral versus central cannulation. Innovations (Phila). (2025) 20(2):148–57. 10.1177/15569845251333344 40261087 PMC12090206

[2] Maj G Regesta T Campanella A Cavozza C Parodi G Audo A. Optimal management of patients treated with minimally invasive cardiac surgery in the era of enhanced recovery after surgery and fast-track protocols: a narrative review. J Cardiothorac Vasc Anesth. (2022) 36(3):766–75. 10.1053/j.jvca.2021.02.035 33840614

[3] Cheng L Zhu H Xing W Fu M Ke Y. Minimal invasive thoracoscopic mitral valve surgery. Braz J Cardiovasc Surg. (2022) 37(5):688–93. 10.21470/1678-9741-2020-0260 36346773 PMC9670354

[4] Liu J Wei P Ma J Fang L Chen Z Cao Z. Propensity-matched analysis of two port approach versus three port approach for totally thoracoscopic mitral valve replacement. J Thorac Dis. (2020) 12(10):5986–95. 10.21037/jtd-20-2901 33209431 PMC7656324

[5] Liu J Chen B Zhang Y-Y Fang L-Z Xie B Huang H-L. Mitral valve replacement via minimally invasive totally thoracoscopic surgery versus traditional median sternotomy: a propensity score matched comparative study. Ann Transl Med. (2019) 7(14):341. 10.21037/atm.2019.07.07 31475211 PMC6694243

[6] Maier RH Kasim AS Zacharias J Vale L Graham R Walker A. Minimally invasive versus conventional sternotomy for mitral valve repair: protocol for a multicentre randomised controlled trial (UK Mini Mitral). BMJ Open. (2021) 11(4):e047676. 10.1136/bmjopen-2020-047676 33853807 PMC8054102

[7] Claessens J Yilmaz A Mostien T Van Genechten S Claes M Packlé L. 90-day patient-centered outcomes after totally endoscopic cardiac surgery: a prospective cohort study. J Clin Med. (2022) 11(9):2674. 10.3390/jcm11092674 35566800 PMC9103144

[8] Salenger R Lobdell K Grant MC. Update on minimally invasive cardiac surgery and enhanced recovery after surgery. Curr Opin Anaesthesiol. (2024) 37(1):10–5. 10.1097/aco.0000000000001322 37865831

[9] Wang D Wang S Wu J Le S Xie F Li X. Nomogram models to predict postoperative hyperlactatemia in patients undergoing elective cardiac surgery. Front Med (Lausanne). (2021) 8:763931. 10.3389/fmed.2021.763931 34926506 PMC8674505

[10] Wang S Wang D Huang X Wang H Le S Zhang J. Development and validation of a nomogram model for early postoperative hyperlactatemia in patients undergoing cardiac surgery. J Thorac Dis. (2021) 13(9):5395–408. 10.21037/jtd-21-1004 34659806 PMC8482329

[11] Govender P Tosh W Burt C Falter F. Evaluation of increase in intraoperative lactate level as a predictor of outcome in adults after cardiac surgery. J Cardiothorac Vasc Anesth. (2020) 34(4):877–84. 10.1053/j.jvca.2019.10.039 31787432

[12] Aksoy R Karakoc AZ Cevirme D Elibol A Yigit F Yilmaz Ü. Predictive factors of prolonged ventilation following cardiac surgery with cardiopulmonary bypass. Braz J Cardiovasc Surg. (2021) 36(6):780–7. 10.21470/1678-9741-2020-0164 34236782 PMC8641772

[13] Chen DX Zhang YY Xiong XL Zhou L Shi J. Association between intraoperative lactate levels and acute kidney injury after on-pump cardiac surgery: a retrospective cohort study across two centers. BMC Surg. (2025) 25(1):324. 10.1186/s12893-025-03082-6 40739501 PMC12308900

[14] Wang X- Bao R Lan Y Zhao Z- Yang X- Wang Y- The incidence, risk factors, and prognosis of acute kidney injury in patients after cardiac surgery. Front Cardiovasc Med. (2024) 11:1396889. 10.3389/fcvm.2024.1396889 39081365 PMC11286402

[15] Nagin DS Jones BL Elmer J. Recent advances in group-based trajectory modeling for clinical research. Annu Rev Clin Psychol. (2024) 20(1):285–305. 10.1146/annurev-clinpsy-081122-012416 38382118

[16] Wei Y Zhuang J Li J Wang Z Wang J Zhang X. Lactate trajectories and outcomes in patients with sepsis in the intensive care unit: group-based trajectory modeling. Front Public Health. (2025) 13:1610220. 10.3389/fpubh.2025.1610220 40589821 PMC12206821

[17] Liang Z Zhao M Liu K Liang W Luo S Guan J. Lactate trajectories in early survivors of sepsis: a new lens on mortality risk. Shock. (2025) 64(4):386–96. 10.1097/shk.0000000000002653 40720292 PMC12435258

[18] Fang Y Zhang Y Shen X Dou A Xie H Zhang Y. Utilization of lactate trajectory models for predicting acute kidney injury and mortality in patients with hyperlactatemia: insights across three independent cohorts. Ren Fail. (2025) 47(1):2474205. 10.1080/0886022x.2025.2474205 40074720 PMC11905305

[19] Zhang H Li Z Zheng W Zhang L Yang T Xie K. Risk stratification of patients with acute respiratory distress syndrome complicated with sepsis using lactate trajectories. BMC Pulm Med. (2022) 22(1):339. 10.1186/s12890-022-02132-6 36071432 PMC9451114

[20] Tariq MA Malik MK Uddin QS Altaf Z Zafar M. Minimally invasive procedure versus conventional redo sternotomy for mitral valve surgery in patients with previous cardiac surgery: a systematic review and meta-analysis. J Chest Surg. (2023) 56(6):374–86. 10.5090/jcs.23.038 37817430 PMC10625962

[21] Manosalva C Quiroga J Hidalgo AI Alarcón P Ansoleaga N Hidalgo MA. Role of lactate in inflammatory processes: friend or foe. Front Immunol. (2022) 12:808799. 10.3389/fimmu.2021.808799 35095895 PMC8795514

[22] Fang Y Li Z Yang L Li W Wang Y Kong Z. Emerging roles of lactate in acute and chronic inflammation. Cell Commun Signal. (2024) 22(1):276. 10.1186/s12964-024-01624-8 38755659 PMC11097486

[23] Squiccimarro E Stasi A Lorusso R Paparella D. Narrative review of the systemic inflammatory reaction to cardiac surgery and cardiopulmonary bypass. Artif Organs. (2022) 46(4):568–77. 10.1111/aor.14171 35061922 PMC9303696

[24] Heo RH Wang MK Meyre PB Birchenough L Park L Vuong K. Associations of inflammatory biomarkers with the risk of morbidity and mortality after cardiac surgery: a systematic review and meta-analysis. Can J Cardiol. (2023) 39(11):1686–94. 10.1016/j.cjca.2023.07.021 37495205

[25] Viikinkoski E Aittokallio J Lehto J Ollila H Relander A Vasankari T. Prolonged systemic inflammatory response syndrome after cardiac surgery. J Cardiothorac Vasc Anesth. (2024) 38(3):709–16. 10.1053/j.jvca.2023.12.017 38220516

[26] Hakim MZ Tewarson V Kumar S Rahul K Prabha R Singh SK. Early mortality in cardiac surgery - is lactate significant? Braz J Cardiovasc Surg. (2024) e20230245:e20230245. 10.21470/1678-9741-2023-0245 39418497 PMC11488391

