# Supplementary material for: Postoperative lactate trajectories predict in-hospital adverse outcomes and differ by surgical approach in cardiac surgery: a propensity score-matched study
Source: Front Cardiovasc Med. 2026 Sep 17;13:1921332. doi: 10.3389/fcvm.2026.1921332 (PMC13627056; doi:10.3389/fcvm.2026.1921332)
Supplement: Supplementary file 1 [file Supplementaryfile1.docx]

# Supplementary Methods

**S1 Lactate Measurement and Sampling Quality Control**

Arterial blood lactate was measured at nine predefined postoperative time points (T0–T8), corresponding to 0, 2, 4, 6, 12, 24, 48, 72, and 120 h after surgery. Acceptable sampling windows were: T0, within 30 min of ICU admission; T1–T3, within ±30 min of the nominal time; T4–T6, within ±3 h; and T7–T8, within ±6 h. Time points were non-equidistantly distributed and were processed as actual elapsed hours throughout all modeling and visualization steps. Lactate concentrations were determined by a point-of-care blood gas analyzer and expressed in mmol L⁻¹. Observed values ranged from 0.6 to 9.9 mmol L⁻¹ across the full cohort; no extreme values were identified, and no truncation was applied.

**S2 Propensity Score Matching Covariates**

All propensity score–matching covariates were measured preoperatively and spanned four domains: (i) demographics—age, sex, and body mass index (BMI); (ii) comorbidities—hypertension, diabetes mellitus, smoking history, atrial fibrillation, coronary artery disease, pulmonary arterial hypertension, and renal insufficiency; (iii) cardiac function—left ventricular ejection fraction (LVEF), left atrial diameter, and New York Heart Association (NYHA) functional class; and (iv) preoperative laboratory parameters—N-terminal pro-B-type natriuretic peptide (NT-proBNP), serum creatinine, hemoglobin, albumin, and prothrombin time–international normalized ratio (PT-INR). Surgical type (valvular, congenital heart disease, or other) was designated a mandatory exact-matching variable.

**S3 Detailed Definitions of Outcome Variables**

Composite endpoints. The components of the three predefined composite endpoints—major adverse events (MAE), major adverse cardiovascular events (MACE), and MACE-Core—and the analyses to which each applies are described in the corresponding tables in the main text.

Secondary outcomes were defined as follows:

- Mechanical ventilation >48 h (MV>48 h): cumulative invasive mechanical ventilation exceeding 48 h from ICU admission to the time of first successful extubation.
- New-onset atrial fibrillation: postoperative atrial fibrillation lasting ≥30 s confirmed in patients with no pre-existing atrial fibrillation.
- Low cardiac output syndrome (LCOS): inotropic support for ≥24 h accompanied by hypotension, oliguria, or peripheral hypoperfusion.
- Re-intubation: tracheal re-intubation for respiratory failure within 7 days of extubation.
- Renal replacement therapy/continuous renal replacement therapy (RRT/CRRT): newly initiated renal replacement therapy for acute kidney injury.
- Re-exploration for hemostasis: surgical re-exploration for bleeding or cardiac tamponade.
- Delirium: positive result on the Confusion Assessment Method for the ICU (CAM-ICU).
- Stroke: new ischemic or hemorrhagic stroke confirmed by cranial computed tomography (CT) or magnetic resonance imaging (MRI).
- Tracheostomy: performed for prolonged mechanical ventilation.
- Intra-aortic balloon pump (IABP) and extracorporeal membrane oxygenation (ECMO): initiated intraoperatively or postoperatively.
- In-hospital death: all-cause mortality during the index hospitalization.
- ICU transfusion: administration of any blood product during the ICU stay.

Continuous outcomes comprised mechanical ventilation duration (h), ICU length of stay (d), and total hospital length of stay (d).

**S4 Statistical Details of Propensity Score Matching**

Matching was conducted in random order using the MatchIt package in R. Pre-matching between-group comparisons were performed using the independent-samples t-test, Mann–Whitney U test, and χ²/Fisher exact test, as appropriate. Post-matching comparisons used the paired t-test, Wilcoxon signed-rank test, and McNemar test. Matching quality was assessed using standardized mean differences (SMDs); an |SMD| < 0.10 was considered indicative of adequate covariate balance. Results were visualized using a Love plot, propensity score distribution density plots, and a variance ratio plot (Figure S1).

**S5 Detailed Specification of Group-Based Trajectory Modelling**

Modeling was implemented using the hlme function of the lcmm package in R. Fixed effects were specified as polynomial functions of elapsed time. No subject-specific random effects were included. Initial parameter values were optimized by grid search. Missing data were handled within the model under full-information maximum likelihood (FIML).

Model selection proceeded in two stages. First, with k fixed at three groups, linear, quadratic, and cubic polynomial specifications were compared; the order minimizing the Bayesian information criterion (BIC) was selected. Second, with the polynomial order fixed, models for k = 1–5 groups were estimated. The optimal group number was determined jointly by information criteria (Akaike information criterion [AIC], BIC, and sample-size-adjusted BIC [SABIC]), classification quality (average posterior probability of assignment [APPA] ≥ 0.70, odds of correct classification [OCC] > 5, and entropy), a minimum group proportion ≥ 5%, and clinical interpretability. For k ≥ 4, the minimum group proportion fell below 5% or clinical interpretability was inadequate; a three-group quadratic polynomial model was therefore selected.

Classification quality of the final model was satisfactory (APPA = 0.901, exceeding the 0.70 threshold), indicating low assignment uncertainty. Bias attributable to modal assignment was considered negligible; a posterior-probability-weighted analysis was therefore not substituted for the primary analysis.

**S6 Detailed Analysis of the Association Between Surgical Approach and Trajectory Membership**

A 2 × 3 contingency table was constructed for surgical approach (TTCS vs. open sternotomy) and lactate trajectory membership (Low-Stable < Moderate-Resolving < Persistently Elevated). Distributions were compared by Pearson chi-squared test. The independent association of TTCS with trajectory membership was estimated globally by proportional-odds ordinal logistic regression. Three tiers of incremental covariate adjustment were applied; T-Model designations in this section apply exclusively to trajectory-membership analyses. T-Model 1 was unadjusted and excluded post-exposure variables to approximate the total association. T-Model 2 adjusted for age, left ventricular ejection fraction (LVEF), cardiopulmonary bypass (CPB) duration, and intraoperative transfusion. T-Model 3 extended T-Model 2 with aortic cross-clamp time, total operative duration, intraoperative blood loss, sex, body mass index (BMI), and surgical type. Cumulative odds ratios (ORs) and 95% confidence intervals (CIs) were reported. The proportional-odds assumption was verified by likelihood-ratio test (LRT); if violated (p < 0.05), multinomial logistic regression with Low-Stable as the reference category was pre-specified as the primary analysis, with ordinal regression results retained as supplementary reference.

Where the proportional-odds assumption was violated (p < 0.05), multinomial logistic regression with Low-Stable as the reference was adopted as the primary analysis. The same three-tier adjustment scheme (T-Models 1–3) was applied. Adjusted ORs and 95% CIs for TTCS were estimated separately for membership in the Moderate-Resolving and Persistently Elevated trajectories; T-Model 2 results were presented in Table 2. Ordinal logistic regression results were retained as supplementary reference (Supplementary Table S3).

Two pre-specified auxiliary analyses were performed, each applying the T-Model 1–3 adjustment tiers. First, a dedicated binary logistic regression was fitted with Persistently Elevated membership as the outcome (Supplementary Table S4). Second, ordinary least-squares (OLS) regression was performed with the log-transformed posterior probability of Persistently Elevated membership [log(P + 1 × 10⁻⁶)] as the continuous dependent variable, to provide an estimate of the a-path coefficient for the mediation analysis. Stratified analyses of Persistently Elevated membership were additionally performed by surgical type (congenital heart disease [CHD], valvular, and other); interaction was assessed by LRT. Based on T-Model 2, with remaining covariates held at their median or mode, adjusted probabilities of membership in each trajectory were predicted for each surgical group. Absolute probability differences were computed to quantify the clinical magnitude.

**S7 Detailed Analysis of the Association Between Lactate Trajectory and In-Hospital Outcomes**

Binary outcomes were compared by Pearson chi-squared test (Fisher's exact test when any expected cell count was < 5) and continuous outcomes by Kruskal–Wallis test, expressed as median (IQR). Trend tests used the Cochran–Armitage test for binary outcomes and Spearman rank correlation for continuous outcomes. Multivariable analyses used Low-Stable as the reference category. Binary outcomes were analyzed by logistic regression reporting adjusted ORs. Continuous outcomes were log-transformed [log(y + 0.5)] and analyzed by log-linear regression reporting geometric mean ratios (GMRs). An ordinal trend term was incorporated, with p_trend reported by Wald test. All analyses were based on complete cases.

Covariate adjustment was pre-specified in tiers according to the events-per-variable (EPV) principle. When the number of events exceeded 100, a full covariate set was used (surgical approach, age, LVEF, CPB duration, and intraoperative transfusion). For 50–100 events, CPB duration and intraoperative transfusion were omitted. For 30–50 events, only surgical approach and age were retained. For fewer than 30 events, only surgical approach was included. Sparse outcomes with fewer than 15 events—tracheostomy, stroke, and extracorporeal membrane oxygenation (ECMO)—were reported with unadjusted ORs only.

For each outcome, three models were fitted: O-Model 1 (univariable), O-Model 2 (EPV-tiered primary model), and O-Model 3 (sensitivity model additionally including BMI, sex, and surgical type, restricted to outcomes with ≥ 100 events). O-Model designations in this section apply exclusively to trajectory–outcome analyses. Under O-Model 2, the covariate scheme was tiered by event count as follows: Primary (> 100 events)—surgical approach, age, LVEF, CPB duration, and intraoperative transfusion; Reduced1 (50–100 events)—as Primary, omitting CPB duration and intraoperative transfusion; Reduced2 (30–50 events)—surgical approach and age only; Minimal (< 30 events)—surgical approach only. All schemes were pre-specified.

Six subgroups were pre-specified for the primary MAE outcome: surgical approach, age (median 38 years), CPB duration (median 90 min), pulmonary arterial hypertension, intraoperative transfusion, and surgical type (CHD vs. valvular and other). Diabetes (n = 16) and LVEF < 50% (n = 3) were excluded owing to insufficient sample size. Within each subgroup, a logistic regression was fitted to estimate the OR for Persistently Elevated vs. Low-Stable trajectory membership, with adjustment following O-Model 2 excluding the stratifying variable itself. Interaction was assessed by LRT, and results were displayed as a forest plot. Discrimination (C-statistic) and calibration (Hosmer–Lemeshow test with 10 equal-frequency probability groups) of the primary MAE model (O-Model 2) were assessed, and Nagelkerke R² was reported.

**S8 Detailed Methods for Exploratory Path Association Analysis**

Temporal precedence between the lactate trajectory sampling window (T0–T120 h) and the constituent events of major adverse events (MAE) could not be established. The analysis was therefore downgraded to an exploratory path association analysis; no causal mediation inference was drawn. The sole analytical objective was to quantify the relative contribution of the indirect association along the "surgical approach → lactate trajectory → MAE" pathway.

The total association (c-path) was estimated by multivariable binary logistic regression using the covariate adjustment scheme specified in the outcome model (O-Model; Supplementary Methods S7). Path decomposition was performed using the mediate() function in the R mediation package with 5,000 nonparametric bootstrap iterations, yielding the average causal mediation effect (ACME; indirect-path estimate), the average direct effect (ADE; direct-path estimate), and the proportion of the total association attributable to each. Ninety-five percent confidence intervals were constructed by the percentile method; p values were two-sided. All effect sizes were reported on the linear probability scale for associative quantification only; no causal interpretation was ascribed.

One primary analysis and two pre-specified sensitivity analyses were conducted; all were adjusted for age, left ventricular ejection fraction (LVEF), cardiopulmonary bypass (CPB) duration, and intraoperative transfusion. In the primary analysis, the log-transformed posterior probability of Persistently Elevated trajectory membership served as a continuous pathway variable (path model: ordinary least squares [OLS] regression; outcome model: binary logistic regression). Sensitivity analysis 1 coded trajectory membership as an ordinal trichotomy (path model: proportional-odds ordinal logistic regression). Sensitivity analysis 2 merged the Moderate-Resolving and Persistently Elevated trajectories into a binary "high-risk trajectory" variable (path model: binary logistic regression). Robustness to unmeasured confounding was evaluated using the medsens() function, which reported the critical confounding correlation ρ* required to nullify the ACME estimate and the ρ value at which the 95% CI of the ACME first included zero. A probit-link outcome model was specified for this test, with all other settings identical to the primary analysis.

**S9 Detailed Methods for Early Lactate Predictive Modelling**

Analyses were restricted to the 1,254 patients from the matched cohort with complete lactate measurements at both T0 and T2h (10 patients were excluded for missing values at one or both time points). Major adverse cardiovascular events (MACE) were defined independently of trajectory modeling and designated as the prediction outcome. This approach precluded circular reasoning and information leakage that would arise from predicting trajectory membership from the early lactate values used in its construction. Candidate predictors were T0 lactate, T2h lactate, and ΔLactate (= T2h − T0, mmol L⁻¹).

Five sequential binary logistic regression models were constructed (the P-Model numbering convention applies exclusively to this section): P-Model 1 included T0 lactate only; P-Model 2 included T2h lactate only; P-Model 3 included both T0 and T2h lactate; P-Model 4 added age, sex, body mass index (BMI), and LVEF; P-Model 5 additionally included surgical approach (total thoracoscopic cardiac surgery [TTCS]) and surgical type. Odds ratios (ORs) and 95% confidence intervals (CIs) were reported per 1 mmol L⁻¹ increment.

Discrimination was assessed by the area under the receiver operating characteristic curve (AUC) with DeLong confidence intervals. Pairwise AUC comparisons were performed using the paired DeLong test; primary comparisons were P-Model 1 versus P-Model 2 and P-Model 2 versus P-Model 3. Five-fold cross-validation was performed for P-Model 5. Calibration was evaluated by the Hosmer–Lemeshow test (10 groups) and visualized with calibration plots. Clinical net benefit was assessed by decision curve analysis (DCA).

Optimal probability thresholds for P-Models 2 and 5 were determined by the Youden index; sensitivity, specificity, positive predictive value (PPV), and negative predictive value (NPV) were reported at each threshold. For P-Model 2, the Youden-optimal probability threshold was back-substituted into the logistic regression equation to derive the corresponding T2h lactate cutoff concentration, reported in Table 4. The diagnostic performance of fixed T2h lactate cutoffs at 2, 3, 4, and 5 mmol L⁻¹ was additionally evaluated. A sensitivity analysis substituting MACE-Core for MACE as the primary outcome was performed for P-Models 1–3. Analyses were conducted using the pROC, ResourceSelection, caret, dcurves, and tidyverse packages in R.

**S10 Detailed Methods for Mechanistic Biomarker Analyses of Inflammatory and Organ-Function Trajectories**

Five biomarkers were selected: interleukin-6 (IL-6), C-reactive protein (CRP), and procalcitonin (PCT) as inflammatory markers, and serum creatinine and N-terminal pro-B-type natriuretic peptide (NT-proBNP) as organ-function markers. IL-6, PCT, serum creatinine, and NT-proBNP were measured at postoperative days (POD) 0–5; CRP was measured at POD 0–4. Trajectory group assignments were derived from maximum posterior probability labels generated by group-based trajectory modeling (GBTM).

A separate linear mixed-effects model was fitted for each biomarker using the lme() function in the R nlme package with an unstructured covariance structure. Fixed effects comprised trajectory group (Low-Stable as reference), POD time point (POD 0 as reference), and a trajectory group × time interaction term; age and LVEF were included as additional covariates. A patient-level random intercept was specified as the random effect. Parameters were estimated by restricted maximum likelihood (REML). Fixed effects were tested by Type III analysis of variance; group main effects, time main effects, and interaction effects were reported.

Post hoc comparisons were performed using the emmeans package to compute estimated marginal means (EMM) and 95% CIs for each trajectory group at each time point. For log-transformed biomarkers (IL-6, CRP, PCT, and NT-proBNP), estimates were back-transformed to geometric means; between-group differences were expressed as ratios. Serum creatinine results were expressed as arithmetic means with mean differences. Pairwise comparisons between trajectory groups at each time point were adjusted for multiple comparisons by Bonferroni correction.
