# Supplementary material for: Postoperative lactate trajectories predict in-hospital adverse outcomes and differ by surgical approach in cardiac surgery: a propensity score-matched study
Source: Front Cardiovasc Med. 2026 Sep 17;13:1921332. doi: 10.3389/fcvm.2026.1921332 (PMC13627056; doi:10.3389/fcvm.2026.1921332)
Supplement: Supplementary file 2 [file Supplementaryfile2.docx]

**Supplementary Tables**

Supplementary Table S1 — GBTM Model Selection

| k | n_params | loglik | AIC | BIC | SABIC | entropy | min_APPA | min_OCC | min_group_pct |
| --- | --- | --- | --- | --- | --- | --- | --- | --- | --- |
| 1 | 4 | -15575.4 | 31158.81 | 31179.38 | 31166.67 | NA | NA | NA | 100 |
| 2 | 8 | -13050.6 | 26117.26 | 26158.39 | 26132.98 | 0.989 | 0.989 | 51.29 | 9.34 |
| 3 | 12 | -12336.9 | 24697.89 | 24759.59 | 24721.47 | 0.883 | 0.901 | 12.66 | 5.62 |
| 4 | 16 | -11890.9 | 23813.75 | 23896.02 | 23845.2 | 0.867 | 0.908 | 11.56 | 2.45 |
| 5 | 20 | -11704.8 | 23449.51 | 23552.35 | 23488.82 | 0.87 | 0.898 | 10.27 | 1.03 |

**Note.** The three-group quadratic model (k = 3) was selected by balancing information criteria (AIC, BIC, SABIC), classification quality (entropy = 0.883; min_APPA = 0.901; min_OCC = 12.66), and clinical interpretability, with all groups exceeding the 5% minimum size threshold. Models with k ≥ 4 produced at least one group comprising <5% of the sample, limiting interpretability. Classification quality indices are undefined (NA) for the one-group solution. AIC, Akaike information criterion; BIC, Bayesian information criterion; SABIC, sample-size-adjusted BIC; APPA, average posterior probability of assignment (per-group minimum); OCC, odds of correct classification (per-group minimum).

Supplementary Table S2 — Descriptive Statistics of Lactate

| time | N | Mean | SD | Median | Q1 | Q3 | Missing |
| --- | --- | --- | --- | --- | --- | --- | --- |
| 0 | 1264 | 1.83 | 0.57 | 1.8 | 1.4 | 2.2 | 0 |
| 2 | 1264 | 2.13 | 0.75 | 2.1 | 1.6 | 2.5 | 10 |
| 4 | 1264 | 2.55 | 1.05 | 2.4 | 1.9 | 2.9 | 10 |
| 6 | 1264 | 2.72 | 1.17 | 2.5 | 2 | 3 | 15 |
| 12 | 1264 | 2.62 | 1.12 | 2.4 | 2 | 2.9 | 23 |
| 24 | 1264 | 2.39 | 1.06 | 2.2 | 1.8 | 2.7 | 32 |
| 48 | 1264 | 2.26 | 0.95 | 2.1 | 1.7 | 2.6 | 57 |
| 72 | 1264 | 2.17 | 0.91 | 2 | 1.6 | 2.5 | 76 |
| 120 | 1264 | 2.12 | 0.88 | 2 | 1.6 | 2.5 | 95 |

**Note.** Time points denote actual elapsed postoperative hours; T0 = ICU/CCU admission. The uniform N of 1,264 across all rows reflects the full matched cohort. Missing values indicate measurements not obtained within the predefined quality-control window (Supplementary Methods S1) and were accommodated by the full-information maximum-likelihood estimator of group-based trajectory modeling. SD, standard deviation; Q1/Q3, first/third quartile.

Supplementary Table S3 — Ordinal Logistic Regression

| Model | Term | OR | LCI | UCI | OR | P |
| --- | --- | --- | --- | --- | --- | --- |
| T-Model1 (Unadjusted) | TTCS | 0.540058 | 0.427767 | 0.681827 | 0.54 (0.43-0.68) | 2.22E-07 |
| T-Model2 (Primary adjusted) | TTCS | 0.581908 | 0.45595 | 0.742662 | 0.58 (0.46-0.74) | 1.36E-05 |
| T-Model 2 (Primary adjusted) | age | 0.997712 | 0.987662 | 1.007864 | 1.00 (0.99-1.01) | 0.657 |
| T-Model 2 (Primary adjusted) | lvef | 1.00005 | 0.970401 | 1.030605 | 1.00 (0.97-1.03) | 0.997 |
| T-Model 2 (Primary adjusted) | cpb | 0.998225 | 0.995296 | 1.001163 | 1.00 (1.00-1.00) | 0.236 |
| T-Model 2 (Primary adjusted) | tx_intra | 1.321128 | 1.039706 | 1.678726 | 1.32 (1.04-1.68) | 0.0227 |
| T-Model 3 (Fully adjusted) | TTCS | 0.575331 | 0.450327 | 0.735035 | 0.58 (0.45-0.74) | 9.74E-06 |
| T-Model 3 (Fully adjusted) | age | 0.997851 | 0.987501 | 1.008311 | 1.00 (0.99-1.01) | 0.686 |
| T-Model 3 (Fully adjusted) | lvef | 0.999113 | 0.969196 | 1.029953 | 1.00 (0.97-1.03) | 0.954 |
| T-Model 3 (Fully adjusted) | cpb | 0.998581 | 0.995639 | 1.001532 | 1.00 (1.00-1.00) | 0.345 |
| T-Model 3 (Fully adjusted) | tx_intra | 1.33658 | 1.049252 | 1.702589 | 1.34 (1.05-1.70) | 0.0188 |
| T-Model 3 (Fully adjusted) | bmi | 0.994576 | 0.978326 | 1.011095 | 0.99 (0.98-1.01) | 0.518 |
| T-Model 3 (Fully adjusted) | male | 1.01245 | 0.789015 | 1.299158 | 1.01 (0.79-1.30) | 0.923 |
| T-Model 3 (Fully adjusted) | surg_typeCHD | 1.157278 | 0.875333 | 1.530037 | 1.16 (0.88-1.53) | 0.305 |
| T-Model 3 (Fully adjusted) | surg_typeOther | 1.455448 | 0.883152 | 2.398601 | 1.46 (0.88-2.40) | 0.141 |

**Note.** Proportional-odds ordinal logistic regression; outcome ordered Low-Stable < Moderate-Resolving < Persistently Elevated. ORs represent cumulative odds for TTCS versus open sternotomy. T-Model 1, unadjusted; T-Model 2, adjusted for age, LVEF, CPB duration, and intraoperative transfusion; T-Model 3, additionally adjusted for BMI, sex, and surgical type. Because the proportional-odds assumption was violated (likelihood-ratio test p = 0.038), multinomial logistic regression (main Table 2) constitutes the primary analysis; these results are provided as supplementary reference only. CPB duration and intraoperative transfusion were classified as post-exposure variables and included to assess over-adjustment. TTCS, total thoracoscopic cardiac surgery; OR, odds ratio; CI, confidence interval; LVEF, left ventricular ejection fraction; CPB, cardiopulmonary bypass; BMI, body mass index.

Supplementary Table S4 — Binary Logistic Regression (Sensitivity Analysis for PE)

| T-Model | term | OR | LCI | UCI | OR | P |
| --- | --- | --- | --- | --- | --- | --- |
| T-Model 1 (Unadjusted) | TTCS | 0.272 | 0.15 | 0.47 | 0.27 (0.15-0.47) | 7.29E-06 |
| T-Model 2 (Primary adjusted) | TTCS | 0.277 | 0.152 | 0.477 | 0.28 (0.15-0.48) | 9.57E-06 |
| T-Model 3 (Full sensitivity) | TTCS | 0.277 | 0.152 | 0.477 | 0.28 (0.15-0.48) | 9.73E-06 |

**Note.** Binary logistic regression; outcome: Persistently Elevated (PE) trajectory membership (reference: Low-Stable + Moderate-Resolving combined). Covariate adjustment follows the same three-tier scheme as Supplementary Table S3. Near-identical ORs across T-Models 1–3 indicate that the protective association of TTCS with PE membership is robust to covariate adjustment. TTCS, total thoracoscopic cardiac surgery; PE, Persistently Elevated; OR, odds ratio; CI, confidence interval.

Supplementary Table 5 — Association Between TTCS and Persistently Elevated Trajectory by Surgical Type

| surg_type | n | Stern_HR_n | TTCS_HR_n | OR | LCI | UCI | OR | P_Fisher |
| --- | --- | --- | --- | --- | --- | --- | --- | --- |
| Valve | 350 | 11/175 (6.3%) | 4/175 (2.3%) | 0.35 | 0.08 | 1.21 | 0.35 (0.08-1.21) | 0.111 |
| CHD | 834 | 43/417 (10.3%) | 10/417 (2.4%) | 0.214 | 0.095 | 0.44 | 0.21 (0.09-0.44) | 2.79E-06 |
| Other | 80 | 1/40 (2.5%) | 2/40 (5.0%) | 2.035 | 0.102 | 124.059 | 2.03 (0.10-124.06) | 1 |

**Note.** OR, odds ratio; CI, confidence interval. Reference category: open sternotomy. ORs were estimated by binary logistic regression within each subgroup. p-values are from two-sided Fisher's exact test. Likelihood-ratio test for surgical type × trajectory interaction: p = 0.163. The "Other" subgroup (n = 80) was underpowered for meaningful interpretation.

Supplementary Table S6 — In-Hospital Outcomes by Lactate Trajectory Group

| Outcome | Low-Stable (n=832) | Moderate-Resolving (n=361) | Persistently-Elevated (n=71) | P (overall) | P (Low vs High) | Test |
| --- | --- | --- | --- | --- | --- | --- |
| MAE (composite) | 29/832 (3.5%) | 46/361 (12.7%) | 27/71 (38.0%) | <0.001 | <0.001 | Chi2 |
| In-hospital mortality | 4/832 (0.5%) | 8/361 (2.2%) | 4/71 (5.6%) | <0.001 | 0.002 | Fisher |
| Mechanical ventilation > 48 h | 39/832 (4.7%) | 64/361 (17.7%) | 36/71 (50.7%) | <0.001 | <0.001 | Chi2 |
| RRT/CRRT | 14/832 (1.7%) | 25/361 (6.9%) | 16/71 (22.5%) | <0.001 | <0.001 | Fisher |
| Low cardiac output syndrome | 8/832 (1.0%) | 15/361 (4.2%) | 9/71 (12.7%) | <0.001 | <0.001 | Fisher |
| New-onset atrial fibrillation | 85/832 (10.2%) | 57/361 (15.8%) | 16/71 (22.5%) | <0.001 | 0.005 | Chi2 |
| Delirium | 27/832 (3.2%) | 28/361 (7.8%) | 11/71 (15.5%) | <0.001 | <0.001 | Fisher |
| Re-exploration for bleeding | 15/832 (1.8%) | 15/361 (4.2%) | 6/71 (8.5%) | 0.002 | 0.004 | Fisher |
| ICU transfusion | 58/832 (7.0%) | 60/361 (16.6%) | 26/71 (36.6%) | <0.001 | <0.001 | Chi2 |
| MV duration (hours) | 11.0 (7.0-18.0) | 18.0 (10.0-32.0) | 49.0 (19.0-113.0) | <0.001 | <0.001 | Kruskal-Wallis |
| Hospital LOS (days) | 15.0 (13.0-19.0) | 16.0 (13.0-20.0) | 17.0 (14.0-21.0) | 0.012 | 0.008 | Kruskal-Wallis |
| ICU LOS (days) | 3.7 (2.4-5.5) | 4.1 (2.9-6.2) | 5.7 (3.0-8.7) | <0.001 | <0.001 | Kruskal-Wallis |

**Note.** Data are n/N (%) or median (IQR). P (overall): chi-squared or Kruskal–Wallis test across all three groups. P (Low vs High): Fisher's exact test or Kruskal–Wallis test comparing Low-Stable versus Persistently Elevated. MAE, major adverse events; RRT, renal replacement therapy; CRRT, continuous RRT; MV, mechanical ventilation; LOS, length of stay; ICU, intensive care unit.

Supplementary Table S7 — Multivariable Logistic Regression of Binary Outcomes by Lactate Trajectory Group

| Outcome | O-Model | Group | N_events | OR | LCI | UCI | OR | P | P_trend |
| --- | --- | --- | --- | --- | --- | --- | --- | --- | --- |
| MAE (composite) | O-Model 1 (Unadjusted) | Moderate-Resolving vs Low-Stable | 102 | 4.043569 | 2.495466 | 6.552061 | 4.04 (2.50-6.55) | 1.40E-08 | 1.76E-20 |
| MAE (composite) | O-Model 1 (Unadjusted) | Persistently-Elevated vs Low-Stable | 102 | 16.99138 | 9.27239 | 31.1362 | 16.99 (9.27-31.14) | 4.87E-20 | 1.76E-20 |
| MAE (composite) | O-Model 2 (Full adjusted) | Moderate-Resolving vs Low-Stable | 102 | 3.788835 | 2.329907 | 6.161306 | 3.79 (2.33-6.16) | 7.90E-08 | 7.51E-18 |
| MAE (composite) | O-Model 2 (Full adjusted) | Persistently-Elevated vs Low-Stable | 102 | 14.7324 | 7.907593 | 27.4475 | 14.73 (7.91-27.45) | 2.38E-17 | 7.51E-18 |
| MAE (composite) | O-Model 3 (Fully adjusted) | Moderate-Resolving vs Low-Stable | 102 | 3.769726 | 2.310932 | 6.149396 | 3.77 (2.31-6.15) | 1.07E-07 | 5.20E-18 |
| MAE (composite) | O-Model 3 (Fully adjusted) | Persistently-Elevated vs Low-Stable | 102 | 16.01181 | 8.49222 | 30.18974 | 16.01 (8.49-30.19) | 1.02E-17 | 5.20E-18 |
| Mechanical ventilation > 48 h | O-Model 1 (Unadjusted) | Moderate-Resolving vs Low-Stable | 139 | 4.381594 | 2.879315 | 6.667684 | 4.38 (2.88-6.67) | 5.32E-12 | 3.03E-27 |
| Mechanical ventilation > 48 h | O-Model 1 (Unadjusted) | Persistently-Elevated vs Low-Stable | 139 | 20.91429 | 11.88092 | 36.81594 | 20.91 (11.88-36.82) | 5.78E-26 | 3.03E-27 |
| Mechanical ventilation > 48 h | O-Model 2 (Full adjusted) | Moderate-Resolving vs Low-Stable | 139 | 4.260982 | 2.78751 | 6.513328 | 4.26 (2.79-6.51) | 2.16E-11 | 3.42E-25 |
| Mechanical ventilation > 48 h | O-Model 2 (Full adjusted) | Persistently-Elevated vs Low-Stable | 139 | 20.78588 | 11.53513 | 37.4554 | 20.79 (11.54-37.46) | 5.58E-24 | 3.42E-25 |
| Mechanical ventilation > 48 h | O-Model 3 (Fully adjusted) | Moderate-Resolving vs Low-Stable | 139 | 4.244382 | 2.771611 | 6.499749 | 4.24 (2.77-6.50) | 2.96E-11 | 1.90E-25 |
| Mechanical ventilation > 48 h | O-Model 3 (Fully adjusted) | Persistently-Elevated vs Low-Stable | 139 | 22.20548 | 12.24133 | 40.28022 | 22.21 (12.24-40.28) | 1.91E-24 | 1.90E-25 |
| New-onset atrial fibrillation | O-Model 1 (Unadjusted) | Moderate-Resolving vs Low-Stable | 158 | 1.647794 | 1.148416 | 2.364322 | 1.65 (1.15-2.36) | 0.0067 | 0.000217 |
| New-onset atrial fibrillation | O-Model 1 (Unadjusted) | Persistently-Elevated vs Low-Stable | 158 | 2.556578 | 1.40277 | 4.659417 | 2.56 (1.40-4.66) | 0.00218 | 0.000217 |
| New-onset atrial fibrillation | O-Model 2 (Full adjusted) | Moderate-Resolving vs Low-Stable | 158 | 1.60382 | 1.114133 | 2.308736 | 1.60 (1.11-2.31) | 0.011 | 0.00077 |
| New-onset atrial fibrillation | O-Model 2 (Full adjusted) | Persistently-Elevated vs Low-Stable | 158 | 2.366151 | 1.280338 | 4.37281 | 2.37 (1.28-4.37) | 0.00598 | 0.00077 |
| New-onset atrial fibrillation | O-Model 3 (Fully adjusted) | Moderate-Resolving vs Low-Stable | 158 | 1.615796 | 1.120652 | 2.329711 | 1.62 (1.12-2.33) | 0.0102 | 0.00087 |
| New-onset atrial fibrillation | O-Model 3 (Fully adjusted) | Persistently-Elevated vs Low-Stable | 158 | 2.322542 | 1.252676 | 4.306141 | 2.32 (1.25-4.31) | 0.00747 | 0.00087 |
| ICU transfusion | O-Model 1 (Unadjusted) | Moderate-Resolving vs Low-Stable | 144 | 2.660099 | 1.810638 | 3.908082 | 2.66 (1.81-3.91) | 6.20E-07 | 1.21E-14 |
| ICU transfusion | O-Model 1 (Unadjusted) | Persistently-Elevated vs Low-Stable | 144 | 7.710345 | 4.441183 | 13.38594 | 7.71 (4.44-13.39) | 3.95E-13 | 1.21E-14 |
| ICU transfusion | O-Model 2 (Full adjusted) | Moderate-Resolving vs Low-Stable | 144 | 2.50467 | 1.698761 | 3.692913 | 2.50 (1.70-3.69) | 3.57E-06 | 1.98E-12 |
| ICU transfusion | O-Model 2 (Full adjusted) | Persistently-Elevated vs Low-Stable | 144 | 6.657118 | 3.785099 | 11.70834 | 6.66 (3.79-11.71) | 4.69E-11 | 1.98E-12 |
| ICU transfusion | O-Model 3 (Fully adjusted) | Moderate-Resolving vs Low-Stable | 144 | 2.520382 | 1.708109 | 3.718922 | 2.52 (1.71-3.72) | 3.20E-06 | 1.99E-12 |
| ICU transfusion | O-Model 3 (Fully adjusted) | Persistently-Elevated vs Low-Stable | 144 | 6.669674 | 3.784593 | 11.75412 | 6.67 (3.78-11.75) | 5.25E-11 | 1.99E-12 |
| Delirium | O-Model 1 (Unadjusted) | Moderate-Resolving vs Low-Stable | 66 | 2.506951 | 1.4553 | 4.318564 | 2.51 (1.46-4.32) | 0.000926 | 1.21E-06 |
| Delirium | O-Model 1 (Unadjusted) | Persistently-Elevated vs Low-Stable | 66 | 5.466049 | 2.585779 | 11.55462 | 5.47 (2.59-11.55) | 8.69E-06 | 1.21E-06 |
| Delirium | O-Model 2 (Reduced1 adjusted) | Moderate-Resolving vs Low-Stable | 66 | 2.491882 | 1.442112 | 4.305821 | 2.49 (1.44-4.31) | 0.00107 | 4.02E-06 |
| Delirium | O-Model 2 (Reduced1 adjusted) | Persistently-Elevated vs Low-Stable | 66 | 5.111648 | 2.372101 | 11.01511 | 5.11 (2.37-11.02) | 3.11E-05 | 4.02E-06 |
| RRT / CRRT | O-Model 1 (Unadjusted) | Moderate-Resolving vs Low-Stable | 55 | 4.347364 | 2.23264 | 8.465122 | 4.35 (2.23-8.47) | 1.54E-05 | 2.60E-13 |
| RRT / CRRT | O-Model 1 (Unadjusted) | Persistently-Elevated vs Low-Stable | 55 | 16.9974 | 7.890453 | 36.61534 | 17.00 (7.89-36.62) | 4.63E-13 | 2.60E-13 |
| RRT / CRRT | O-Model 2 (Reduced1 adjusted) | Moderate-Resolving vs Low-Stable | 55 | 4.043861 | 2.069198 | 7.902972 | 4.04 (2.07-7.90) | 4.37E-05 | 2.38E-11 |
| RRT / CRRT | O-Model 2 (Reduced1 adjusted) | Persistently-Elevated vs Low-Stable | 55 | 13.96134 | 6.359708 | 30.64906 | 13.96 (6.36-30.65) | 4.99E-11 | 2.38E-11 |
| Re-exploration for bleeding | O-Model 1 (Unadjusted) | Moderate-Resolving vs Low-Stable | 36 | 2.361272 | 1.141804 | 4.883151 | 2.36 (1.14-4.88) | 0.0205 | 0.000499 |
| Re-exploration for bleeding | O-Model 1 (Unadjusted) | Persistently-Elevated vs Low-Stable | 36 | 5.027692 | 1.88726 | 13.39385 | 5.03 (1.89-13.39) | 0.00124 | 0.000499 |
| Re-exploration for bleeding | O-Model 2 (Reduced2 adjusted) | Moderate-Resolving vs Low-Stable | 36 | 2.266243 | 1.091853 | 4.703799 | 2.27 (1.09-4.70) | 0.0281 | 0.00145 |
| Re-exploration for bleeding | O-Model 2 (Reduced2 adjusted) | Persistently-Elevated vs Low-Stable | 36 | 4.455056 | 1.634926 | 12.13971 | 4.46 (1.63-12.14) | 0.00349 | 0.00145 |
| Low cardiac output syndrome | O-Model 1 (Unadjusted) | Moderate-Resolving vs Low-Stable | 32 | 4.465318 | 1.876 | 10.6285 | 4.47 (1.88-10.63) | 0.00072 | 3.20E-08 |
| Low cardiac output syndrome | O-Model 1 (Unadjusted) | Persistently-Elevated vs Low-Stable | 32 | 14.95161 | 5.57391 | 40.10663 | 14.95 (5.57-40.11) | 7.76E-08 | 3.20E-08 |
| Low cardiac output syndrome | O-Model 2 (Reduced2 adjusted) | Moderate-Resolving vs Low-Stable | 32 | 4.393671 | 1.839442 | 10.49467 | 4.39 (1.84-10.49) | 0.000863 | 1.29E-07 |
| Low cardiac output syndrome | O-Model 2 (Reduced2 adjusted) | Persistently-Elevated vs Low-Stable | 32 | 14.2467 | 5.159044 | 39.34224 | 14.25 (5.16-39.34) | 2.96E-07 | 1.29E-07 |
| IABP | O-Model 1 (Unadjusted) | Moderate-Resolving vs Low-Stable | 21 | 3.922127 | 1.414671 | 10.87397 | 3.92 (1.41-10.87) | 0.00862 | 6.45E-05 |
| IABP | O-Model 1 (Unadjusted) | Persistently-Elevated vs Low-Stable | 21 | 10.42929 | 3.100797 | 35.07812 | 10.43 (3.10-35.08) | 0.000152 | 6.45E-05 |
| IABP | O-Model 2 (Minimal adjusted) | Moderate-Resolving vs Low-Stable | 21 | 3.848465 | 1.382074 | 10.71627 | 3.85 (1.38-10.72) | 0.0099 | 0.000141 |
| IABP | O-Model 2 (Minimal adjusted) | Persistently-Elevated vs Low-Stable | 21 | 9.847457 | 2.836093 | 34.19225 | 9.85 (2.84-34.19) | 0.000317 | 0.000141 |
| In-hospital mortality | O-Model 1 (Unadjusted) | Moderate-Resolving vs Low-Stable | 16 | 4.691218 | 1.403566 | 15.67972 | 4.69 (1.40-15.68) | 0.0121 | 0.000184 |
| In-hospital mortality | O-Model 1 (Unadjusted) | Persistently-Elevated vs Low-Stable | 16 | 12.35821 | 3.022958 | 50.52181 | 12.36 (3.02-50.52) | 0.000466 | 0.000184 |
| In-hospital mortality | O-Model 2 (Minimal adjusted) | Moderate-Resolving vs Low-Stable | 16 | 4.212254 | 1.253978 | 14.14944 | 4.21 (1.25-14.15) | 0.02 | 0.00111 |
| In-hospital mortality | O-Model 2 (Minimal adjusted) | Persistently-Elevated vs Low-Stable | 16 | 9.109509 | 2.179303 | 38.07784 | 9.11 (2.18-38.08) | 0.00247 | 0.00111 |

**Note.** Reference category: Low-Stable trajectory. Values are adjusted OR (95% CI). O-Model 1: unadjusted. O-Model 2/3: progressively adjusted; covariate tiers selected by the events-per-variable principle (details in Supplementary Methods S6). p-trend: ordinal trend test across trajectory groups. IABP, intra-aortic balloon pump; RRT, renal replacement therapy; CRRT, continuous RRT; MAE, major adverse events.

Supplementary Table S8 — Log-Linear Regression of Continuous Outcomes by Lactate Trajectory Group

| Outcome | O-Model | Group | exp_beta | LCI | UCI | Effect_text | P | P_trend |
| --- | --- | --- | --- | --- | --- | --- | --- | --- |
| MV duration (hours) | O-Model 1 (Unadjusted) | Moderate-Resolving vs Low-Stable | 1.635847 | 1.474674 | 1.814636 | 1.64 (1.47-1.81) | 5.55E-20 | 2.22E-43 |
| MV duration (hours) | O-Model 1 (Unadjusted) | Persistently-Elevated vs Low-Stable | 3.653571 | 2.980892 | 4.478049 | 3.65 (2.98-4.48) | 7.56E-34 | 2.22E-43 |
| MV duration (hours) | O-Model 2 (Primary adjusted) | Moderate-Resolving vs Low-Stable | 1.598885 | 1.441761 | 1.773132 | 1.60 (1.44-1.77) | 1.90E-18 | 1.46E-39 |
| MV duration (hours) | O-Model 2 (Primary adjusted) | Persistently-Elevated vs Low-Stable | 3.470764 | 2.828831 | 4.258368 | 3.47 (2.83-4.26) | 3.43E-31 | 1.46E-39 |
| Hospital LOS (days) | O-Model 1 (Unadjusted) | Moderate-Resolving vs Low-Stable | 1.031676 | 0.996356 | 1.068247 | 1.03 (1.00-1.07) | 0.0793 | 0.00301 |
| Hospital LOS (days) | O-Model 1 (Unadjusted) | Persistently-Elevated vs Low-Stable | 1.100401 | 1.027714 | 1.178229 | 1.10 (1.03-1.18) | 0.00611 | 0.00301 |
| Hospital LOS (days) | O-Model 2 (Primary adjusted) | Moderate-Resolving vs Low-Stable | 1.020662 | 0.986481 | 1.056027 | 1.02 (0.99-1.06) | 0.239 | 0.0365 |
| Hospital LOS (days) | O-Model 2 (Primary adjusted) | Persistently-Elevated vs Low-Stable | 1.071554 | 1.001768 | 1.146202 | 1.07 (1.00-1.15) | 0.0443 | 0.0365 |
| ICU LOS (days) | O-Model 1 (Unadjusted) | Moderate-Resolving vs Low-Stable | 1.148611 | 1.073308 | 1.229196 | 1.15 (1.07-1.23) | 6.46E-05 | 8.85E-09 |
| ICU LOS (days) | O-Model 1 (Unadjusted) | Persistently-Elevated vs Low-Stable | 1.382508 | 1.210311 | 1.579205 | 1.38 (1.21-1.58) | 1.99E-06 | 8.85E-09 |
| ICU LOS (days) | O-Model 2 (Primary adjusted) | Moderate-Resolving vs Low-Stable | 1.126199 | 1.054571 | 1.202691 | 1.13 (1.05-1.20) | 0.000402 | 2.00E-07 |
| ICU LOS (days) | O-Model 2 (Primary adjusted) | Persistently-Elevated vs Low-Stable | 1.341141 | 1.177742 | 1.527208 | 1.34 (1.18-1.53) | 1.01E-05 | 2.00E-07 |

**Note.** Reference category: Low-Stable trajectory. Values are geometric mean ratios (GMR, 95% CI) derived from log-linear regression on log-transformed outcomes. O-Model 1: unadjusted; O-Model 2: adjusted for preoperative covariates (details in Supplementary Methods S6). p-trend: ordinal trend test across trajectory groups. MV, mechanical ventilation; LOS, length of stay; ICU, intensive care unit.

Supplementary Table S9 — Multivariable Logistic Regression: Total Association of TTCS with MAE Across Covariate Adjustment Models

| O-Model | Term | OR | LCI | UCI | OR | P |
| --- | --- | --- | --- | --- | --- | --- |
| O-Model 1 (Unadjusted) — total effect | ttcs | 0.449517 | 0.292276 | 0.691352 | 0.45 (0.29-0.69) | 0.000272 |
| O-Model 2 (Primary adjusted) — total effect | ttcs | 0.50773 | 0.323265 | 0.797457 | 0.51 (0.32-0.80) | 0.00326 |
| O-Model 3 (Fully adjusted) — total effect | ttcs | 0.474303 | 0.300806 | 0.747868 | 0.47 (0.30-0.75) | 0.00133 |

**Note.** OR, odds ratio; CI, confidence interval; TTCS, total thoracoscopic cardiac surgery; MAE, major adverse events. O-Model 1: unadjusted. O-Model 2: adjusted for preoperative demographics, comorbidities, cardiac function, and laboratory parameters. O-Model 3: additionally adjusted for intraoperative variables (CPB duration, aortic cross-clamp time, operative duration, and blood loss/transfusion). All estimates represent the total-effect association of TTCS with MAE.

Supplementary Table S10 — Incidence of MACE Component Events in the Matched Cohort

| Event | N | Percent |
| --- | --- | --- |
| In-hospital death | 16 | 1.30% |
| Low cardiac output syndrome (LCOS) | 31 | 2.50% |
| IABP | 21 | 1.70% |
| ECMO | 3 | 0.20% |
| Renal replacement therapy (RRT) | 55 | 4.40% |
| Stroke | 13 | 1.00% |
| Reintubation | 26 | 2.10% |
| Mechanical ventilation > 48h | 139 | 11.10% |
| Reoperation for bleeding | 36 | 2.90% |

**Note.** N = 1,254 (10 patients excluded for missing early lactate data). LCOS, low cardiac output syndrome; IABP, intra-aortic balloon pump; ECMO, extracorporeal membrane oxygenation; RRT, renal replacement therapy. Component events are not mutually exclusive.

Supplementary Table S11 — Sequential Logistic Regression Models for MACE Prediction Using Early Postoperative Lactate

| P-Model | Term | Estimate | SE | OR | OR_LCI | OR_UCI | OR | P |
| --- | --- | --- | --- | --- | --- | --- | --- | --- |
| P-Model 1: T0 alone | (Intercept) | -2.5776 | 0.251 | 0.076 | 0.046 | 0.123 | 0.076 (0.046–.123) | 0 |
| P-Model 1: T0 alone | lact_T0 | 0.6875 | 0.1236 | 1.989 | 1.564 | 2.54 | 1.989 (1.564–.540) | 0 |
| P-Model 2: T2h alone | (Intercept) | -3.1952 | 0.2397 | 0.041 | 0.025 | 0.065 | 0.041 (0.025–.065) | 0 |
| P-Model 2: T2h alone | lact_T2h | 0.8568 | 0.0984 | 2.356 | 1.949 | 2.867 | 2.356 (1.949–.867) | 0 |
| P-Model 3: T0 + T2h (Primary) | (Intercept) | -3.4749 | 0.2943 | 0.031 | 0.017 | 0.054 | 0.031 (0.017–.054) | 0 |
| P-Model 3: T0 + T2h (Primary) | lact_T0 | 0.2384 | 0.1405 | 1.269 | 0.964 | 1.673 | 1.269 (0.964–.673) | 0.0899 |
| P-Model 3: T0 + T2h (Primary) | lact_T2h | 0.7805 | 0.1077 | 2.183 | 1.773 | 2.705 | 2.183 (1.773–.705) | 0 |
| P-Model 4: T0+T2h + Preop Covariates | (Intercept) | -4.1337 | 1.3742 | 0.016 | 0.001 | 0.235 | 0.016 (0.001–.235) | 0.0026 |
| P-Model 4: T0+T2h + Preop Covariates | lact_T0 | 0.2305 | 0.1413 | 1.259 | 0.955 | 1.662 | 1.259 (0.955–.662) | 0.103 |
| P-Model 4: T0+T2h + Preop Covariates | lact_T2h | 0.8158 | 0.109 | 2.261 | 1.831 | 2.809 | 2.261 (1.831–.809) | 0 |
| P-Model 4: T0+T2h + Preop Covariates | age | 0.0168 | 0.0064 | 1.017 | 1.004 | 1.03 | 1.017 (1.004–.030) | 0.0084 |
| P-Model 4: T0+T2h + Preop Covariates | sex | 0.3061 | 0.1515 | 1.358 | 1.008 | 1.826 | 1.358 (1.008–.826) | 0.0433 |
| P-Model 4: T0+T2h + Preop Covariates | bmi | -0.0513 | 0.0208 | 0.95 | 0.911 | 0.989 | 0.950 (0.911–.989) | 0.0136 |
| P-Model 4: T0+T2h + Preop Covariates | lvef | 0.0168 | 0.0186 | 1.017 | 0.98 | 1.055 | 1.017 (0.980–.055) | 0.3678 |
| P-Model 5: P-Model 4 + TTCS + Surgery type | (Intercept) | -3.9445 | 1.3885 | 0.019 | 0.001 | 0.292 | 0.019 (0.001–.292) | 0.0045 |
| P-Model 5: P-Model 4 + TTCS + Surgery type | lact_T0 | 0.2224 | 0.1429 | 1.249 | 0.944 | 1.654 | 1.249 (0.944–.654) | 0.1197 |
| P-Model 5: P-Model 4 + TTCS + Surgery type | lact_T2h | 0.7781 | 0.1098 | 2.177 | 1.761 | 2.709 | 2.177 (1.761–.709) | 0 |
| P-Model 5: P-Model 4 + TTCS + Surgery type | age | 0.0154 | 0.0065 | 1.016 | 1.003 | 1.029 | 1.016 (1.003–.029) | 0.0182 |
| P-Model 5: P-Model 4 + TTCS + Surgery type | sex | 0.2747 | 0.1544 | 1.316 | 0.971 | 1.78 | 1.316 (0.971–.780) | 0.0752 |
| P-Model 5: P-Model 4 + TTCS + Surgery type | bmi | -0.0521 | 0.0209 | 0.949 | 0.911 | 0.988 | 0.949 (0.911–.988) | 0.0126 |
| P-Model 5: P-Model 4 + TTCS + Surgery type | lvef | 0.0195 | 0.0187 | 1.02 | 0.983 | 1.058 | 1.020 (0.983–.058) | 0.2975 |
| P-Model 5: P-Model 4 + TTCS + Surgery type | is_TTCS | -0.5196 | 0.1469 | 0.595 | 0.445 | 0.792 | 0.595 (0.445–.792) | 0.0004 |
| P-Model 5: P-Model 4 + TTCS + Surgery type | surgery_typeOther | 0.4622 | 0.2833 | 1.588 | 0.898 | 2.737 | 1.588 (0.898–.737) | 0.1028 |
| P-Model 5: P-Model 4 + TTCS + Surgery type | surgery_typeValve | 0.0844 | 0.1659 | 1.088 | 0.784 | 1.502 | 1.088 (0.784–.502) | 0.6109 |

**Note.** N = 1,254. Lactate was entered as a continuous variable (per 1 mmol/L increment). MACE, major adverse cardiovascular events; OR, odds ratio; SE, standard error; CI, confidence interval; BMI, body mass index; LVEF, left ventricular ejection fraction; TTCS, total thoracoscopic cardiac surgery.

Supplementary Table S12 — Pairwise AUC Comparisons Among Early Lactate Predictive Models

| Comparison | AUC_A | AUC_B | Delta_AUC | Z_stat | P_value |
| --- | --- | --- | --- | --- | --- |
| P-Model 1 vs P-Model 2 | 0.6095 | 0.6655 | -0.056 | -2.7195 | 0.0065 |
| P-Model 1 vs P-Model 3 | 0.6095 | 0.671 | -0.0615 | -3.5296 | 0.0004 |
| P-Model 2 vs P-Model 3 | 0.6655 | 0.671 | -0.0055 | -1.3426 | 0.1794 |

**Note.** Comparisons were performed using the DeLong method. A positive ΔAUC indicates that Model B outperforms Model A. AUC, area under the receiver operating characteristic curve.

Supplementary Table S13 — Model Discrimination for MACE-Core: Sensitivity Analysis

| P-Model | AUC | AUC_LCI | AUC_UCI | AUC |  |
| --- | --- | --- | --- | --- | --- |
| P-Model 1c: T0 alone (MACE-Core) | 0.6292 | 0.5776 | 0.6808 | 0.629 (0.578–0.681) | |
| P-Model 2c: T2h alone (MACE-Core) | 0.6735 | 0.6189 | 0.7281 | 0.674 (0.619–0.728) | |
| P-Model 3c: T0 + T2h (MACE-Core) | 0.6835 | 0.6291 | 0.738 | 0.684 (0.629–0.738) | |

**Note.** AUC, area under the receiver operating characteristic curve; CI, confidence interval. MACE-Core, composite restricted to severe cardiovascular and cerebrovascular events. N = 1,254.

Supplementary Table S14 — Logistic Regression Coefficients for MACE-Core Predictive Models (Sensitivity Analysis)

| P-Model | Term | Estimate | SE | OR | OR_LCI | OR_UCI | OR | P |
| --- | --- | --- | --- | --- | --- | --- | --- | --- |
| MACE-Core P-Model 1: T0 | (Intercept) | -3.7084 | 0.3481 | 0.025 | 0.012 | 0.048 | 0.025 (0.012–0.048) | 0 |
| MACE-Core P-Model 1: T0 | lact_T0 | 0.7924 | 0.1648 | 2.209 | 1.602 | 3.058 | 2.209 (1.602–3.058) | 0 |
| MACE-Core P-Model 2: T2h | (Intercept) | -4.025 | 0.3028 | 0.018 | 0.01 | 0.032 | 0.018 (0.010–0.032) | 0 |
| MACE-Core P-Model 2: T2h | lact_T2h | 0.8021 | 0.1167 | 2.23 | 1.78 | 2.814 | 2.230 (1.780–2.814) | 0 |
| MACE-Core P-Model 3: T0+T2h | (Intercept) | -4.4204 | 0.3869 | 0.012 | 0.006 | 0.025 | 0.012 (0.006–0.025) | 0 |
| MACE-Core P-Model 3: T0+T2h | lact_T0 | 0.3297 | 0.1901 | 1.391 | 0.958 | 2.019 | 1.391 (0.958–2.019) | 0.0828 |
| MACE-Core P-Model 3: T0+T2h | lact_T2h | 0.698 | 0.131 | 2.01 | 1.559 | 2.606 | 2.010 (1.559–2.606) | 0 |

**Note.** Sensitivity analysis using MACE-Core as the outcome (N = 1,254). OR, odds ratio; SE, standard error; CI, confidence interval. MACE-Core definition detailed in Supplementary Methods S9.

Supplementary Table S15 —Linear Mixed-Effects Model Results: Trajectory Group × Time Interaction for Postoperative Biomarkers

| Marker | P_group | P_time | P_interaction |
| --- | --- | --- | --- |
| IL-6 | 0 | 0 | 0 |
| CRP | 4.08E-07 | 0 | 0 |
| PCT | 0.116566 | 0 | 0 |
| Creatinine | 0.962133 | 0 | 0 |
| NT-ProBNP | 0.00228 | 0 | 0 |

**Note**. p-values are derived from linear mixed-effects models with trajectory group, time, and group × time interaction as fixed effects and patient as a random effect. IL-6, interleukin-6; CRP, C-reactive protein; PCT, procalcitonin; NT-proBNP, N-terminal pro-B-type natriuretic peptide.

**Supplementary Figure S1.** **Propensity score matching diagnostics.** (A) Love plot of absolute standardized mean differences (|SMD|) before and after 1:1 nearest-neighbor matching (caliper = 0.2); dashed line, |SMD| = 0.10 threshold. (B) Variance ratios for continuous covariates; dashed lines, 0.5 and 2.0 bounds. (C) Propensity score kernel density distributions before and after matching. All covariates achieved |SMD| < 0.10 post-matching.

**Supplementary Figure S2. GBTM model-selection diagnostics.** (A) Information criteria (AIC, BIC, SABIC) by number of trajectory classes (k). (B) Classification quality indices at each k; dotted line, 0.70 acceptability threshold. (C) Smallest class proportion by k; dashed line, 5% minimum. Criteria jointly supported k = 3 (entropy = 0.883, min-APPA = 0.901, min-OCC = 12.66). AIC, Akaike information criterion; APPA, average posterior probability of assignment; BIC, Bayesian information criterion; OCC, odds of correct classification; SABIC, sample-size-adjusted BIC.

**Supplementary Figure S3. Postoperative continuous outcomes by lactate trajectory.** Box plots of mechanical ventilation duration, ICU length of stay, and hospital length of stay across the Low-Stable, Moderate-Resolving, and Persistently Elevated trajectory groups. Medians are annotated within boxes. p-values are from Kruskal–Wallis tests.

**Supplementary Figure S4. ρ-sensitivity analysis for the indirect-path estimate.** Indirect-path estimates (y-axis) as a function of residual mediator–outcome error correlation ρ, indexing unmeasured confounding magnitude and direction. Vertical reference at ρ = 0; shaded bands, 95% CI. (A) Open sternotomy group (X = 0); null-crossing at ρ ≈ −0.05. (B) TTCS group (X = 1); null-crossing at ρ ≈ +0.05 to +0.10. The critical ρ* of 0.05–0.10 is readily attributable to unmeasured hemodynamic status; all path-decomposition results should be regarded as exploratory.

**Supplementary Figure S5. Early lactate distribution by MACE status.** Violin plots with embedded box plots comparing (A) T0 lactate, (B) T2h lactate, and (C) ΔLactate between Non-MACE and MACE groups. Box boundaries, IQR; center line, median. MACE, major adverse cardiovascular events.
